# Interleukin-1β signaling in fenestrated capillaries is sufficient to trigger sickness responses in mice

**DOI:** 10.1186/s12974-017-0990-7

**Published:** 2017-11-09

**Authors:** J. Gabriel Knoll, Stephanie M. Krasnow, Daniel L. Marks

**Affiliations:** Department of Pediatrics, Papé Family Pediatric Research Institute, Oregon Health & Science University, Mail Code L481 3181 SW Sam Jackson Park Rd, Portland, OR 97239 USA

**Keywords:** Sickness behavior, Inflammation, Fenestrated capillaries, Cytokine, Hypothalamus, Endothelial cells, Microglia

## Abstract

**Background:**

The physiological and behavioral symptoms of sickness, including fever, anorexia, behavioral depression, and weight loss can be both beneficial and detrimental. These sickness responses are triggered by pro-inflammatory cytokines acting on cells within the brain. Previous research demonstrates that the febrile response to peripheral insults depends upon prostaglandin production by vascular endothelial cells, but the mechanisms and specific cell type(s) responsible for other sickness responses remain unknown. The purpose of the present study was to identify which cells within the brain are required for sickness responses triggered by central nervous system inflammation.

**Methods:**

Intracerebroventricular (ICV) administration of 10 ng of the potent pro-inflammatory cytokine interleukin-1β (IL-1β) was used as an experimental model of central nervous system cytokine production. We examined which cells respond to IL-1β in vivo via fluorescent immunohistochemistry. Using multiple transgenic mouse lines expressing Cre recombinase under the control of cell-specific promoters, we eliminated IL-1β signaling from different populations of cells. Food consumption, body weight, movement, and temperature were recorded in adult male mice and analyzed by two-factor ANOVA to determine where IL-1β signaling is essential for sickness responses.

**Results:**

Endothelial cells, microglia, ependymal cells, and astrocytes exhibit nuclear translocation of NF-κB (nuclear factor kappa-light-chain-enhancer of activated B cells) in response to IL-1β. Interfering with IL-1β signaling in microglia, endothelial cells within the parenchyma of the brain, or both did not affect sickness responses. Only mice that lacked IL-1β signaling in all endothelium including fenestrated capillaries lacked sickness responses.

**Conclusions:**

These experiments show that IL-1β-induced sickness responses depend on intact IL-1β signaling in blood vessels and suggest that fenestrated capillaries act as a critical signaling relay between the immune and nervous systems.

**Trial registration:**

Not applicable.

**Electronic supplementary material:**

The online version of this article (10.1186/s12974-017-0990-7) contains supplementary material, which is available to authorized users.

## Background

The biological response to illness and injury is an evolutionarily conserved phenomenon that promotes survival. Many disparate conditions result in the same combination of physiologic and behavioral symptoms including changes to metabolic status, fever, behavioral depression, and anorexia (collectively, the sickness response) [1]. As each of these symptoms is regulated at least in part by elements of the central nervous system (CNS), it is clear that there is a regulated, coordinated CNS response to disease. While there is growing understanding of the specific neural circuits that are involved in regulating individual attributes such as appetite [2] and body temperature [3], less is known about the specific cell types and signaling pathways that initiate the sickness response.

Outside of the CNS, production of cytokines is the predominant mechanism for coordinating the response to a wide variety of infectious pathogens and also plays a key role in the response to traumatic injury, organ failure, and neurodegenerative diseases [4–7]. Pro-inflammatory cytokines are released into the general circulation by peripheral immune cells to signal the presence of disease, stimulate inflammation, and promote healing [8]. Cytokines, particularly interleukin-1β (IL-1β), also act at the level of the brain to trigger changes in neuronal activity that results in sickness responses. Intracerebroventricular (ICV) administration of 10 ng IL-1β causes fever, anorexia, lethargy, and weight loss without raising circulating levels of IL-1β, and sickness responses triggered by peripheral inflammation are blocked by ICV administration of the endogenous IL-1 receptor antagonist (Il1ra) [9–11]. Although neuronal activity is ultimately responsible for controlling physiologic and behavioral set points, disruption of IL-1β signaling in neurons, astrocytes, and oligodendrocytes does not affect the sickness response [12]. This suggests that another cell type is responsible for modulating neuronal activity in response to inflammation.

Vascular endothelial cells, which comprise the wall of blood vessels and form an integral component of the blood-brain barrier (BBB) that prevents IL-1β from passively diffusing into the brain, represent one example of cellular signaling intermediaries between peripheral inflammation and the CNS [13, 14]. As both the conduit that carries inflammatory signals to the brain and the barricade that impedes free diffusion into the CNS, endothelial cells play multiple roles in regulating the sickness response to peripheral disease. In response to peripheral IL-1β, endothelial cells produce prostaglandins (PGs), lipid soluble signaling molecules that are not restricted by the BBB [15, 16]. This is particularly relevant to the febrile response, as interfering with IL-1β signaling or PG synthesis in endothelial cells reduces fever without altering other sickness responses [17, 18]. This implies that other mechanisms are also involved in regulating the sickness response.

In addition to causing increased circulating cytokine levels, peripheral diseases that result in sickness responses also cause the production of pro-inflammatory cytokines, including IL-1β, within the CNS. Disease models of bacterial and viral infection, cancer, and organ failure all cause increased IL-1β within the brain, presenting an additional common pathway for generating sickness responses [10, 19–21]. IL-1β is produced by microglia and perivascular macrophages in and around the circumventricular organs (CVOs) and choroid plexus (ChP), leading to site-specific amplification of peripheral inflammatory signals and elevated IL-1β in the cerebrospinal fluid (CSF) which can act via volume transmission on deeper, BBB-isolated brain regions [22–24]. ICV injections of small amounts of IL-1β achieve CSF concentrations similar to that caused by peripheral insults, providing a model for studying how sickness responses are triggered by this shared pathway [25, 26]. Despite considerable effort, the precise cellular targets of CNS-produced IL-1β necessary for the sickness response remain unidentified.

We examined the specific cellular components of the CNS that are required for the sickness response in mice using a model of increased central IL-1β concentration, as is commonly found in response to peripheral inflammation [27]. We hypothesized that blood vessels are critical cellular components in this phase of the CNS inflammation cascade and that disruption of IL-1β signaling in endothelial cells would alter the sickness response. Furthermore, because microglia act to amplify inflammatory signaling within the CNS, we hypothesized that eliminating microglial IL-1β signaling would decrease the severity or duration of sickness responses. Using immunohistochemistry (IHC) following ICV administration of IL-1β, we identified which cell types demonstrate the earliest observable response. We then utilized Cre recombinase-expressing, transgenic mice to systematically disrupt IL-1β signaling in specific cell populations. Our results demonstrate that the physiological and behavioral responses to IL-1β are not derived from cerebral vasculature in general but instead depend upon a relatively small number of fenestrated capillaries located primarily in circumventricular structures.

## Methods

### Animals

For this series of experiments, the following animal strains were used: C57BL/6J (Jackson Laboratory, Bar Harbor, Maine; stock #000664), *Myd88* knockout (*Myd88*KO; Jackson Laboratory stock #009088), *Tek*-Cre (Jackson Laboratory stock #004128), conditional *Myd88* (*Myd88* ^fl/fl^; Jackson Laboratory stock #008888), Rosa26-flox-stop-TdTomato (Jackson Laboratory stock #007908), *Slco1c1*-CreERT2 (provided by Dr. Marcus Schwaninger) [17], *Cx3cr1*-CreERT2 (provided by Dr. Wen-Biao Gan) [28], and conditional IL-1 receptor (*Il1r1* ^fl/fl^ provided by Dr. Randy D. Blakely) [29]. Each of the Cre strains was backcrossed onto the *Myd88* ^fl/fl^ background for at least three generations with the Cre allele carried by the paternal line (father was Cre +/−:*Myd88* ^fl/fl^, and mother was Cre −/−:*Myd88* ^fl/fl^). All experiments were conducted using Cre +/−:*Myd88* ^fl/fl^ as the experimental group and Cre −/−:*Myd88* ^fl/fl^ littermates as the control group. *Cx3cr1*-CreERT2 animals were also bred to the conditional *Il1r1* strain using the same breeding and control strategies.

All animals were housed at Oregon Health & Science University (OHSU) in plastic cages with paper pellet bedding and environmental enrichments on a 12:12-h light/dark cycle in a room maintained at 22 °C. For transgenic strains carrying one of the tamoxifen inducible Cre transgenes, both experimental and control groups were given two treatments of 10 mg tamoxifen (dissolved in sesame oil at a concentration of 100 mg/ml; Sigma-Aldrich, St. Louis, Missouri; Cat#T5648) by oral gavage with 48 h between treatments. Animals were treated with tamoxifen 1 week prior to cannulation and emitter implantation.

### Experimental design

In order to reduce overall animal use and to provide internal controls, a crossover experimental design was employed whereby each animal received both vehicle and IL-1β with at least 3 days between treatments. Group sizes were determined empirically based on previous research that demonstrated that the effects of treatment are large enough (generally greater than 40%) that statistical difference is detected using 5–6 animals per group. Most experiments were first conducted in pilot experiments using three animals per group. These experiments were then replicated, when possible, using a larger number of animals. All data presented are from single experiments, and therefore, the results presented reflect biological replicates (individual animals receiving the same treatment within a single experiment). Data from all animals used in a given experiment were included in all analysis; no animals were excluded.

### Surgical procedures

During all surgical procedures, mice were kept deeply anesthetized with isoflurane. Mice were placed on a stereotactic alignment system (Kopf Instruments, Tujunga, California; model 1900), and a scalp incision was made to expose the skull. Stereotactic coordinates were zeroed at the point where the tip of the cannula touched bregma. Cannula were emplaced in the lateral ventricle to a depth of − 2.25 mm through a 0.5-mm-diameter hole drilled at (*x*, *y*) = (1.0 mm, − 0.5 mm) and secured with dental acrylic (Yates & Bird, Chicago, Illinois; Cat#44118). For behavioral experiments, each animal was also implanted with a transponder (G2 E-Mitter; Starr Life Sciences, Oakmont, Pennsylvania; Cat #870-0010-01) in the peritoneal cavity to monitor core body temperature and movement. Following surgery, animals were individually housed in cages on telemetry platforms (ER4000 Energizer/Receiver; Starr Life Sciences) and allowed to recover for at least 1 week prior to experimentation. During recovery, animals were monitored for fever and movement and were required to reach pre-operative body weight in order to be included.

### Time course of IL-1β-induced inflammation

Male C57Bl/6J mice with permanent cannula implanted into the lateral ventricle were treated with either 1 μl artificial cerebrospinal fluid (aCSF; vehicle) or 1 μl 10 ng/μl recombinant mouse interleukin 1-beta (IL-1β; 10 ng total in aCSF; R&D Systems, Minneapolis, Minnesota; Cat#401-ML-005) and sacrificed at 15 min, 30 min, 90 min, 2 h, 4 h or 8 h post-treatment. For co-localization experiments, the 30-min time point was selected as this represented the time of maximal NF-κB nuclear localization and allowed for consistent, large-scale experiments. At the time of sacrifice, each animal was deeply anesthetized with a mixture of ketamine, acepromazine and xylazine, flushed with 0.01 M phosphate-buffered saline (PBS; pH = 7.4) to remove blood and then perfused with ice cold 4% paraformaldehyde (PFA) in 0.01 M PBS. Brains were dissected free from the skull, post-fixed overnight in 4% PFA while shaking at 4 °C, cryoprotected by immersion in 30% sucrose in 0.01 M PBS overnight at 4 °C, then frozen on dry ice, and stored at − 80 °C.

### Behavioral experiments

For 3 days prior to experimentation, animals were handled and restrained and cannula caps were removed to acclimate the animals to handling stress. In the evening of experimentation, each animal was restrained and injected with either 1 μl aCSF or 1 μl 10 ng/μl IL-1β immediately before lights out (6 pm). Core body temperature and movement counts were automatically recorded every 5 min by the E-Mitter system. Food was manually weighed every 2 h throughout the dark phase and at 12 and 24 h post-treatment. Body weight was recorded at the time of treatment and at 12 and 24 h post-treatment. Following experimentation, each animal was sacrificed and tissue harvested as described in the previous section.

### Immunohistochemistry (IHC)

Cryopreserved frozen brains were sectioned at 30 μm and collected in three serial sets on a sliding microtome (Leica Biosystems, Buffalo Grove, Illinois; SM2000R) equipped with a freezing stage (Physitemp Instruments, Clifton, New Jersey; BFS-5MP). Brain sections were first washed with three changes of PBS then pretreated by quenching with 1% glycine (Sigma-Aldrich, Cat#G8898) in PBS and de-fixing in 0.05% sodium borohydride (Sigma-Aldrich, Cat#71321) in PBS, with three PBS washes between pretreatments. Sections were then blocked in 5% normal goat serum (NGS; Sigma-Aldrich) and 1% hydrogen peroxide in PBS containing 0.3% triton X-100 (Tx; Sigma-Aldrich, Cat#X100) for 30 min at room temperature. Sections were incubated with primary antibodies overnight shaking at 4 °C in PBS containing 1% bovine serum albumin (Sigma-Aldrich, Cat#A2153) and 0.3% Tx. On the following day, sections were washed with three changes of PBS containing 1% NGS and 0.3% Tx and then incubated with secondary antibodies for 2 h at room temperature while shaking in the dark. Sections were counterstained with DAPI at 1:5000 to label nuclear DNA for the final 5 min of secondary incubation, washed with three changes of PBS, and then mounted on gelatin-coated glass slides and coverslipped using Aqua Poly/Mount (Polysciences, Warrington, Pennsylvania; Cat#18606).

The following primary antibodies were used for immunolocalization: rabbit anti-NF-κB (Cell Signaling, Danvers, Massachusetts; Cat#8242; 1:1000), rat anti-Cd31 (BD Biosciences, San Jose, California; Cat#550274; 1:100), rabbit anti-Iba1 (Wako Pure Chemicals, Osaka, Japan; Cat#019-19741; 1:500), chicken anti-vimentin (EMD Millipore, Billerica, Massachusetts; Cat#AB5733; 1:10,000), mouse anti-NeuN (EMD Millipore Cat#MAB377; 1:1000), mouse anti-GFAP (EMD Millipore cat. #MAB360; 1:10,000), rat anti-mouse CD11b (eBioscience, San Diego, California; Cat#14-0112; 1:1000), and chicken anti-GFP (Abcam, Cambridge, Massachusetts; Cat#ab13970; 1:1000). Antibody specificity was verified by omission of primary antibody. Primary antibodies were visualized with the following secondary antibodies: Alexa Fluor 488 goat anti-rabbit IgG (Molecular Probes, Eugene, Oregon; Cat#A-11008), Alexa Fluor 555 goat anti-rabbit IgG (Molecular Probes Cat#A-21428), Alexa Fluor 555 goat anti-rat IgG (Molecular Probes Cat#A-21434), and Alexa Fluor 555 goat anti-chicken IgY (Molecular Probes Cat#A-21437). All secondary antibodies were used at a concentration of 1:500. Observation of TdTomato expression was through native fluorescence only, with no immunoreactivity (IR) amplification. Confocal images were acquired using a Nikon Eclipse inverted microscope equipped with a Nikon A1 confocal image acquisition system (Nikon Instruments, Melville, New York). Epifluorescent images used for supplemental figures were captured on a Leica DM4 microscope (Leica Microsystems) equipped with a DFC340X digital camera (Leica Microsystems) driven by LASv3 software.

### Statistical analysis

For quantification of NF-κB IHC, the entire sets of brain sections (containing every third section) were examined under high magnification. Epifluorescent images of matching sections containing each region of interest were captured using a ×10 objective. Image files were assigned random names, and NF-κB+ nuclei (defined as clear puncta of NF-κB IR overlying DAPI fluorescence) contained within the region of interest were counted in one representative section per animal by an investigator blind to the treatment and/or genotype. For the bilateral paraventricular nucleus (PVN) and arcuate nucleus (ARC), cell counts included both sides of the nucleus. Individual counts of NF-κB+ nuclei from each region were analyzed by two-tailed *t* test with Welch’s correction for unequal variance between aCSF- and IL-1β-treated animals.

The effect of the various conditional knockouts on nuclear localization of NF-κB was assessed by quantification of dual-label IHC. Because of the high cellular density within the CVOs and the close association of multiple cell types (including pericytes, perivascular macrophages, and astrocytes) with brain vasculature, only nuclei that were completely surrounded by the co-label (CD31 or CD11b) were counted when comparing the effect of genotype on individual cell types. Individual cell counts from the PVN and ARC of IL-1β-treated control and knockout animals were analyzed by one-way ANOVA with Tukey’s multiple comparisons between individual genotypes. Cell counts for the PVN were compared between control (*Myd88* ^fl/fl^), *Tek*Δ*Myd88*, and *Slco1c1*Δ*Myd88*. Cell counts for the arcuate nucleus/median eminence (ARC/ME) were compared between control (*Myd88* ^fl/fl^), *Tek*Δ*Myd88*, and *Cx3cr1*Δ*Myd88.* In a separate analysis, cell counts for NF-κB+ microglia in the ARC/ME were compared between *Il1r1* ^fl/fl^ (Cre− control) and *Cx3cr1*Δ*Il1r1* by two-tailed *t* test with Welch’s correction for unequal variance.

Movement counts (voluntary locomotor activity, VLA) and core body temperature (CBT) measurements for individual animals were recorded by the E-Mitter system at 5-min intervals. Change in core body temperature (ΔCBT) was calculated by subtracting the baseline CBT (average of recorded CBT for 4 h prior to treatment) from the measured CBT for each animal at each time point. For 24-h profiles, VLA was summed over each hour to give total counts per hour and ΔCBT values were averaged to give average hourly ΔCBT. For group-wise comparisons of the effects of IL-1β treatment, VLA counts were summed for 6-h blocks (6 pm–12 am) for each animal for total movement analysis, while ΔCBT values were averaged over the 2-h blocks of peak fever (10 pm–12 am). Food intake was calculated from bi-hourly manual food-weight measurements, and changes in body weight were calculated by subtracting the body weight at the time of treatment from body weight measurements taken 12 h after treatment. Statistical analysis was carried out using Prism 6.0 (GraphPad Software, San Diego, California). All physiological data were analyzed by two-way ANOVA for treatment and genotype with Tukey’s multiple comparisons for each group (treatment and genotype) relative to all other groups.

## Results

### Vascular heterogeneity in the brain

**Fig. 1:**
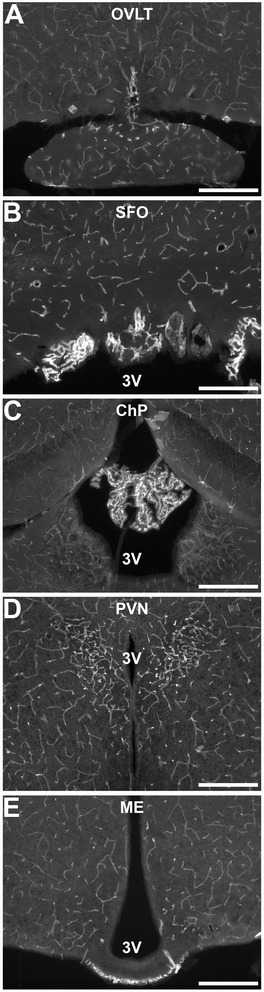
Cd31 immunoreactivity (IR) shows brain vascular heterogeneity. Increased vascular density demonstrated by Cd31 IR in representative epifluorescent images of the organum vasculosum lamina terminalis (OVLT, **a**), subfornical organ (SFO, **b**), choroid plexus (ChP, **c**), paraventricular nucleus (PVN, **d**), and arcuate nucleus/median eminence (ARC/ME, **e**). 3V third ventricle. Scale bars = 500 μm

The distribution of blood vessels in the mouse brain is not uniform. For immunolocalization experiments, we focused on five regions: the organum vasculosum lamina terminalis (OVLT), the subfornical organ (SFO), the choroid plexus (ChP), the paraventricular nucleus (PVN), and the arcuate nucleus/median eminence (ARC/ME; Fig. 1a–e). Cluster of differentiation 31 (Cd31, also known as platelet endothelial cell adhesion molecule, PECAM) IR demonstrates the relatively high vascularity in each of these regions of interest. For greater anatomical context, low-magnification, digital photo-montages of Cd31 IR in whole coronal brain sections are available as a supplement (Additional file 1: Figure S1).

In the three circumventricular organs (CVOs)—the OVLT, SFO, and ME—and in the ChP, the increased vascular density is due to loops of fenestrated capillaries which contribute to a less restrictive BBB in these structures [30–32]. Because of this, the CVOs have long been considered likely sites of entry for circulating cytokines to access the CNS and cause sickness responses [33]. All four structures that contain fenestrated capillaries have also been directly implicated in the neural response to inflammation [34–38]. The PVN contains non-fenestrated, BBB-isolated vessels at a density ~3–5 times greater than most of the brain [39]. The PVN also contains the cell bodies of neurons that control the hypothalamic-pituitary-adrenal axis, modulate the autonomic nervous system, and regulate the muscle catabolism that occurs during disease-associated wasting [10, 40, 41].

### Centrally administered IL-1β activates diverse cells throughout the brain

IL-1β exerts its influence predominantly through binding to a receptor complex located in the cell membrane that includes the IL-1 receptor 1 (Il1r1) and the IL-1 receptor accessory protein (Il1rap) [42]. Binding of IL-1β initiates a sequence of events which requires the adaptor protein Myd88 (myeloid differentiation primary response gene 88) for downstream signaling and IL-1β-induced sickness responses [12, 43–45]. Canonical IL-1β signaling culminates in activation and nuclear translocation of the transcription factor NF-κB (nuclear factor kappa-light-chain-enhancer of activated B cells), resulting in changes in gene transcription [46].

**Fig. 2:**
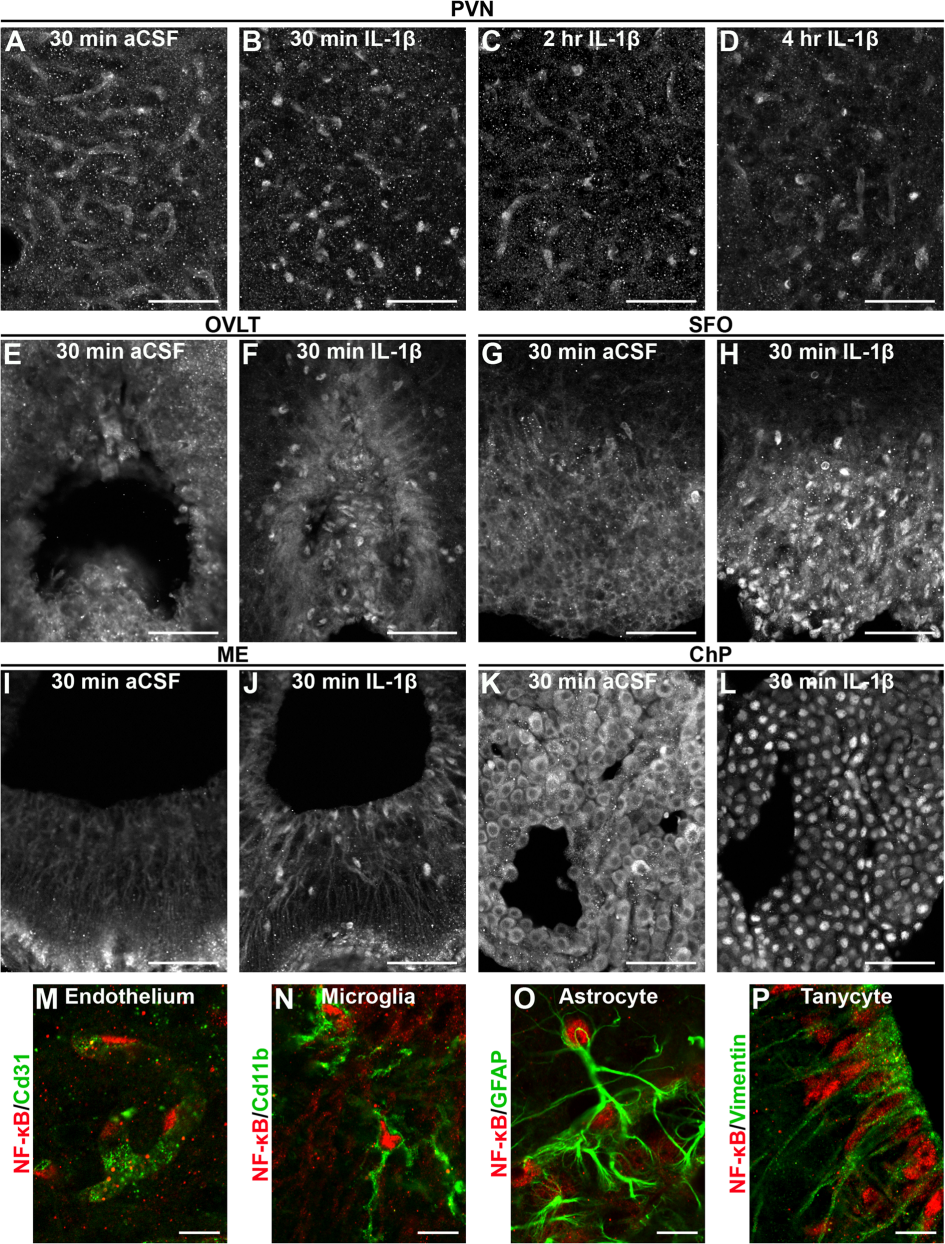
Intracerebroventricular (ICV) administration of IL-1β induces NF-κB nuclear localization in a time- and region-dependent manner. The effect of ICV IL-1β is both rapid and transient as demonstrated by NF-κB immunoreactivity (IR) in representative confocal images of the paraventricular nucleus (PVN). IR in vehicle (aCSF, *n* = 6)-treated animals showed cytoplasmic labeling of blood vessels (**a**). IL-1β treatment caused NF-κB nuclear localization that peaked by 30 min (**b**, *n* = 8), persisted for at least 2 h (**c**, *n* = 4) and returned to near baseline, with cytoplasmic vascular patterns reappearing, by 4 h after treatment (**d**, *n* = 4). This effect was most prominent in and around the PVN, organum vasculosum lamina terminalis (OVLT; **e**, **f**), subfornical organ (SFO; **g**, **h**), arcuate nucleus/median eminence (ARC/ME; **i**, **j**), and choroid plexus (ChP; **k**, **l**). High-magnification confocal images of co-labeling with cell-specific markers shows that vascular endothelial cells (**m**), microglia (**n**), astrocytes (**o**), and tanycytes (**p**) directly respond to IL-1β. Scale bars: **a**–**l** = 50 μm, **m**–**p** = 10 μm

To determine the sites of IL-1β initiated signaling, we examined NF-κB IR in brain sections from animals sacrificed at various times after intracerebroventricular (ICV) administration of either artificial cerebrospinal fluid (aCSF, vehicle) or 10 ng IL-1β in aCSF (Fig. 2). Sections from vehicle-treated animals showed only diffuse, cytoplasmic fluorescence, most evident in blood vessels and ependyma at all times examined (Fig. 2a). No fluorescence above background was detected in sections incubated without primary antibody (Additional file 2: Figure S2A). Sections from IL-1β-treated mice displayed punctate concentrations of NF-κB IR and a marked decrease in cytoplasmic labeling, most notable as a decrease in clearly defined periventricular vascular patterns (Fig. 2b). Co-labeling nuclear DNA with DAPI confirms that these areas of increased NF-κB IR represent IL-1β-induced nuclear translocation (Additional file 3: Figure S3). Analysis of the number of NF-κB+ nuclei revealed a significant effect of treatment in all five regions examined (total NF-κB+ nuclei: OVLT, 0 ± 0 vs. 220.3 ± 37.17, *p* = 0.027; SFO, 2 ± 1.155 vs. 163 ± 36.53, *p* = 0.047; PVN, 7 ± 2.517 vs. 357.3 ± 17.37, *p* = 0.0021; ChP, 2.333 ± 2.333 vs. 518.7 ± 77.23, *p* = 0.022; ARC/ME, 5 ± 2.646 vs. 277.7 ± 10.2, *p* = 0.0008; mean ± SEM for aCSF vs. IL-1β, *n* = 3 for all). This change in NF-κB IR is both rapid and transient, with nuclear labeling evident at 15 min post-treatment, peaking by 30 min, persisting for at least 2 h and returning to baseline, and cytoplasmic labeling by 4 h post-treatment (Fig. 2b–d and Additional file 2: Figure S2C). IL-1β-induced nuclear NF-κB IR was observed scattered throughout the brain with the highest density in regions immediately adjacent to the ventricles including the five regions of interest (Fig. 2e–l). Brain sections from IL-1β-treated *Myd88*KO animals do not exhibit nuclear NF-κB IR at any time examined, indicating that the nuclear translocation of NF-κB as detected by IHC is dependent on expression of functional Myd88 (Additional file 2: Figure S2E).

The high cellular density of the brain (see Additional file 3: Figure S3) and close proximity of multiple different cell types complicates the task of determining exactly which cells contain nuclear NF-κB. This is particularly true of blood vessels within the brain which are intimately associated with pericytes, perivascular macrophages, microglia, and astrocytes. Without cell-type-specific nuclear antigens, such as NeuN for neurons, it is impossible to demonstrate co-localization of nuclear NF-κB with antibodies that label cell membrane or cytoskeletal proteins. In order to determine which cell types respond to IL-1β, we examined multiple different antigens under high magnification and only counted a positive identification as one where the cell-specific marker completely surrounded nuclear NF-κB.

Dual-label IHC revealed nuclear NF-κB IR in vascular endothelium (Cd31+), microglia (cluster of differentiation molecule 11b, Cd11b+), ependymal cells, including some tanycytes (vimentin+), and astrocytes (glial fibrillary acidic protein, GFAP+; Fig. 2m–p). Notably, nuclear NF-κB IR was not observed in neurons, including those within the PVN (Additional file 4: Figure S4). For each of the identified cell types, it was possible to find examples of cells that did not respond to IL-1β, frequently adjacent to examples that did respond. For this reason, subsequent analyses of endothelium focus on the PVN and those of microglia focus on the ARC/ME. In addition to having higher densities of the respective cell type, there tended to be a higher proportion of cells within these regions that respond to IL-1β.

### Recombinase reporter delineates strain-specific genetic recombination

**Fig. 3:**
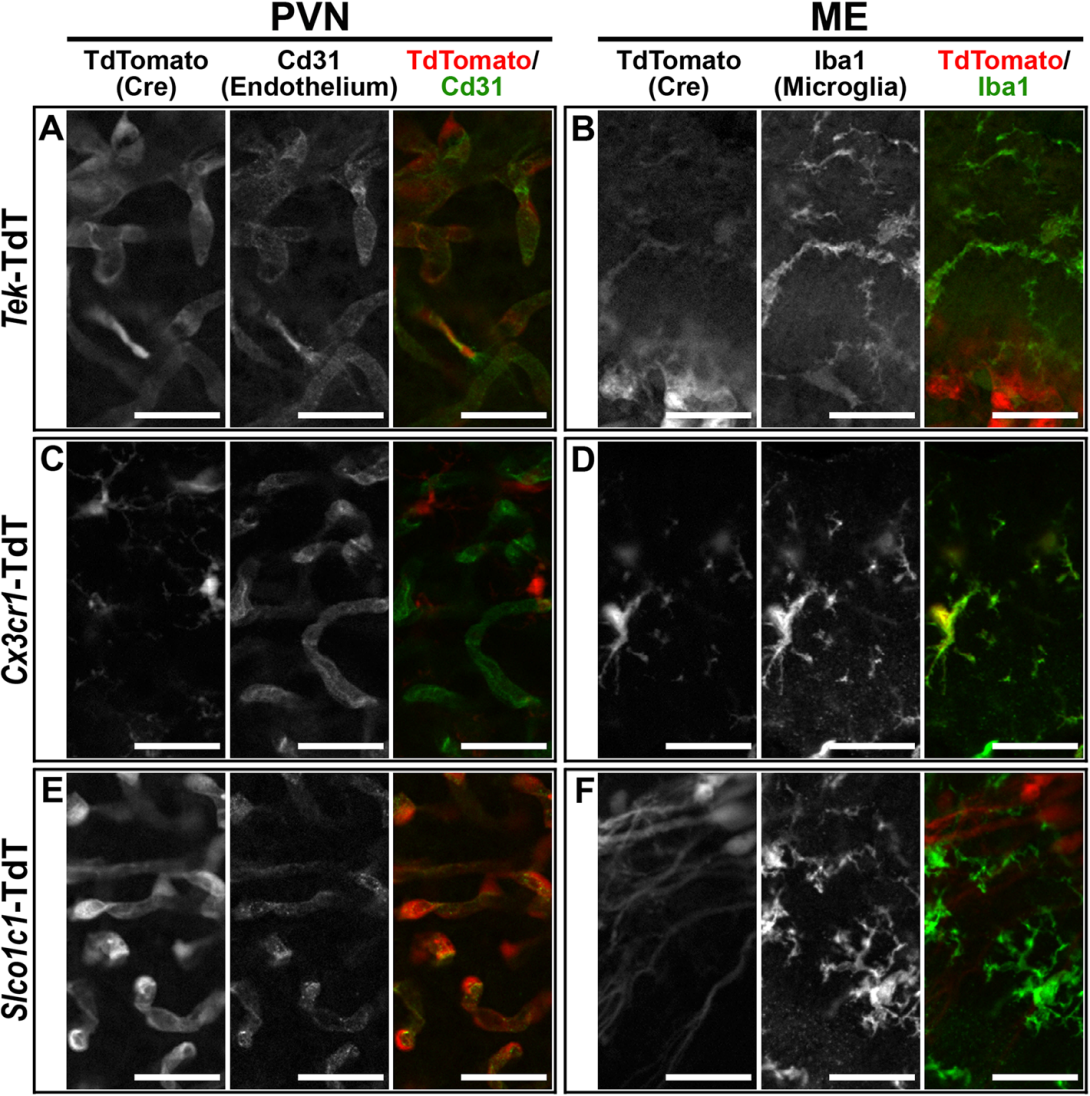
Recombinase reporter shows strain-specific Cre expression. Representative confocal images demonstrate that *Tek*-Cre is expressed in Cd31+ vascular endothelial cells (**a**) and Iba1+ microglia (**b**, *n* = 4). *Cx3cr1*-CreERT2 is only expressed in microglia in both the PVN and ME (**c**, **d**, *n* = 6). *Slco1c1*-CreERT2 is expressed in blood vessels (**e**) but not microglia (**f**, *n* = 4). TdTomato+ cells with long processes in **f** are β1 tanycytes. Scale bars = 25 μm

For this series of experiments, we used three transgenic mouse strains that express Cre recombinase under the control of specific promoters. To assess the contribution of blood vessels to the IL-1β-induced sickness response, we utilized a strain of mice expressing Cre recombinase under the control of the promoter for the angiopoietin-1 receptor, *Tek* (also known as Tie2; *Tek*-Cre) [47]. To determine sites of *Tek*-driven Cre expression, we crossed the *Tek*-Cre and Rosa26-flox-stop-TdTomato (TdT) strains to generate mice that express fluorescent TdT only in cells that express *Tek*-Cre (*Tek*-TdT) [48]. Examination of co-localization of reporter expression and various cell-specific markers in brain sections from *Tek*-TdT animals revealed genetic recombination in all vascular endothelium (Cd31 IR) and all microglia (immunoreactive for ionized calcium-binding adapter molecule-1, Iba1 IR; Fig. 3a, b). This result is in agreement with previously published descriptions of *Tek*-Cre expression in endothelium and myeloid cells [49, 50]. Reporter expression was absent from neurons, astrocytes, and oligodendrocytes (data not shown). This pattern of expression was consistent in all brain regions examined (Additional file 5: Figure S5 A-D).

As the resident immune cells of the brain, microglia are likely candidates for playing a role in the brain response to immune signaling. We investigated the function of microglia in the sickness response using a line of mice that express tamoxifen-inducible Cre and yellow fluorescent protein (YFP) under the control of the endogenous promoter for the fractalkine receptor, *Cx3cr1* (*Cx3cr1*-CreERT2) [28]. This allows for temporal control of Cre activity and an endogenous marker of cells that express the transgene. We verified recombination by crossing the *Cx3cr1*-CreERT2 and TdT lines (*Cx3cr1-*TdT) and found TdT expression exclusively in all microglia in brain sections from tamoxifen-treated animals (Fig. 3c, d). This result demonstrates that *Cx3cr1*-CreERT2 animals can be used to manipulate microglia without affecting blood vessels.

To examine the role of endothelium independent of microglia, we used a line of mice that express tamoxifen-inducible Cre under the control of the promoter for the thyroid hormone transporter and solute carrier organic anion transporter family member 1C1 (*Slco1c1*-CreERT2) [17]. In brain sections from tamoxifen-treated *Slco1c1*-TdT animals, TdT expression was evident in multiple cell types including cuboidal cells of the ChP, a subset of β1 tanycytes [51], some hippocampal neurons, and an unidentified cell type with morphology consistent with GFAP-negative astrocytes (Fig. 3f and Additional file 5: Figure S5). Importantly, TdT expression was observed in all vascular endothelium within the tissue of the brain itself (parenchymal endothelium; Fig. 3e and Additional file 5: Figure S5E–H). Reporter expression was absent from microglia, oligodendrocytes and GFAP+ astrocytes (Fig. 3f and data not shown). This suggests that the *Slco1c1*-CreERT2 line can be used to drive recombination in parenchymal endothelium without affecting microglia.

**Fig. 4:**
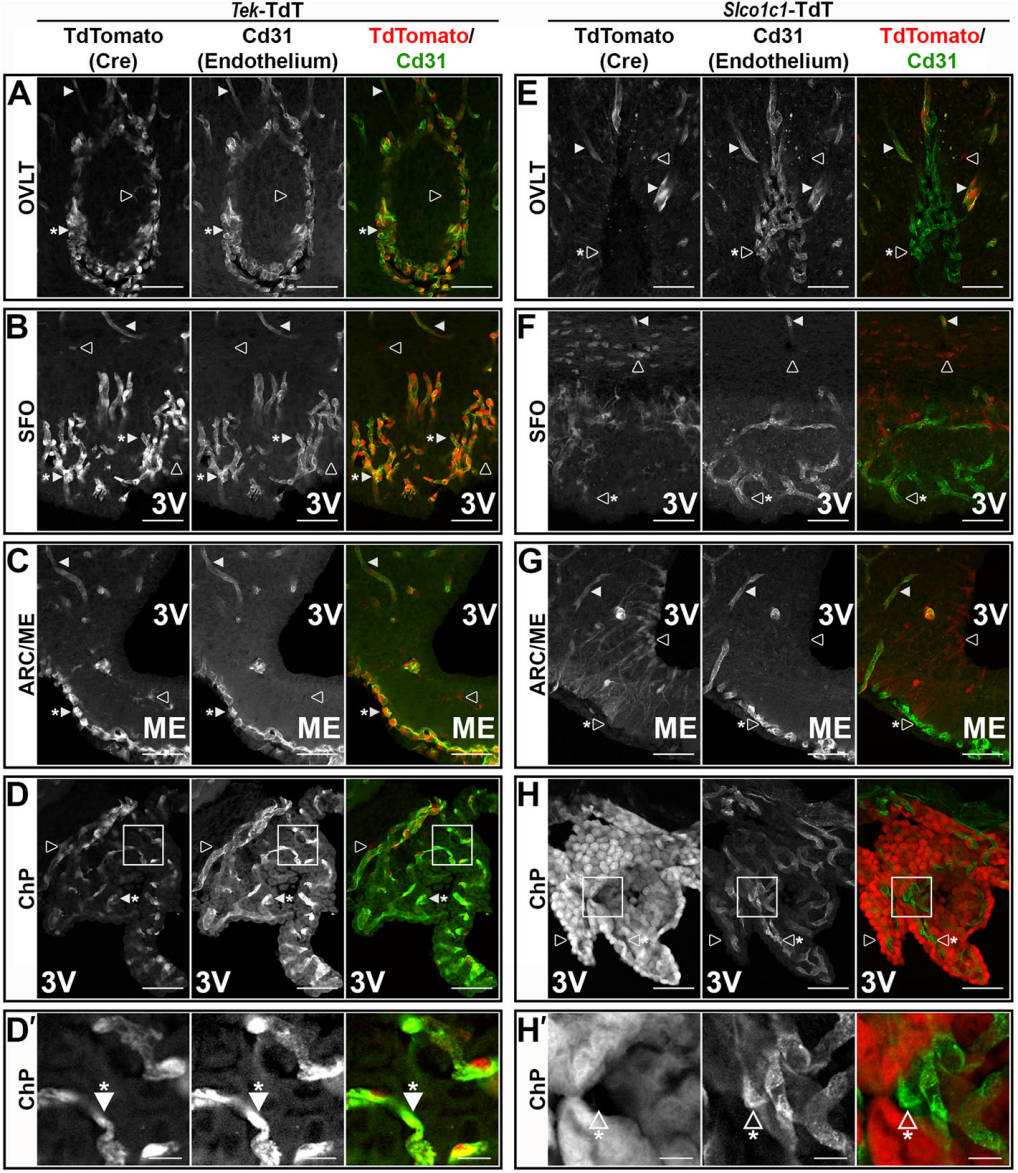
*Tek*-Cre, but not *Slco1c1*-CreERT2, is expressed in fenestrated capillaries of circumventricular organs. Representative confocal images of co-localization of TdTomato (TdT, red) and Cd31 (green) in *Tek-*TdT animals (**a**–**c**, *n* = 4) confirms co-expression in parenchymal endothelium (filled arrowheads) and fenestrated capillaries (filled arrowheads with asterisk) in the organum vasculosum lamina terminalis (OVLT, **a**), subfornical organ (SFO, **b**), arcuate nucleus/median eminence (ARC/ME, **c**), and choroid plexus (ChP, **d**). Reporter expression is also seen in microglia in each of these regions (open arrowheads in **a**–**d**). Co-labeling of TdT (red) and Cd31(green) in *Slco1c1-*TdT animals (**e**–**h**, *n* = 4) shows Cre activity in all parenchymal endothelium (filled arrowheads), but not fenestrated capillaries (open arrowheads with asterisk). Reporter expression is also seen in other cells that do not express Cd31, particularly the cuboidal cells of the choroid plexus (open arrowheads in **e**–**h**). Higher magnification of the boxed regions in **d** and **h** (**d**′ and **h**′) demonstrate that ChP fenestrated capillaries express TdT in *Tek*-Cre but not *Slco1c1*-CreERT2 animals (filled and open arrowheads with asterisk, respectively). Scale bars: **a**–**h** 50 μm; **d**′ and **h**′ 10 μm

While comparing reporter expression with Cd31 IR in *Slco1c1*- and *Tek*-TdT animals, we noted a major difference between the two lines in their vascular expression pattern: *Tek*-Cre causes recombination in all endothelium including fenestrated capillaries (asterisk in Fig. 4A–D), while *Slco1c1*-CreERT2 causes recombination in all parenchymal endothelium but not in fenestrated capillaries (asterisk in Fig. 4E–H). This is especially obvious in the OVLT, SFO, and ME, where the fenestrated capillaries of *Slco1c1-*TdT clearly do not express TdT. Although the high level of reporter expression in ChP cuboidal cells makes it difficult to determine whether recombination occurs in the underlying fenestrated capillaries, when viewed in cross section, there does not appear to be expression in the fenestrated capillary within the tube of ensheathing cuboidal cells (asterisk in Fig. 4H). At higher magnification, the difference in ChP fenestrated capillary reporter expression between *Tek*-TdT and *Slco1c1*-TdT is more obvious (Fig. 4D′, H′). This comparison also highlights the fact that reporter expression occurs outside of endothelium in both lines (open arrowheads in Fig. 4).

### NF-κB IR confirms Cre-dependent IL-1β signaling disruption

To disrupt IL-1β signaling in specific cell populations, we utilized the conditional *Myd88* strain (*Myd88* ^fl/fl^) to generate promoter-specific *Myd88* knockout mice (Δ*Myd88*). By crossing the *Myd88* ^fl/fl^ and the three Cre strains, we produced mice that lack Myd88-dependent IL-1β signaling in all endothelium and microglia (*Tek*Δ*Myd88*), in all microglia alone (*Cx3cr1*Δ*Myd88*), and in parenchymal endothelium but not microglia (*Slco1c1*Δ*Myd88*).

**Fig. 5:**
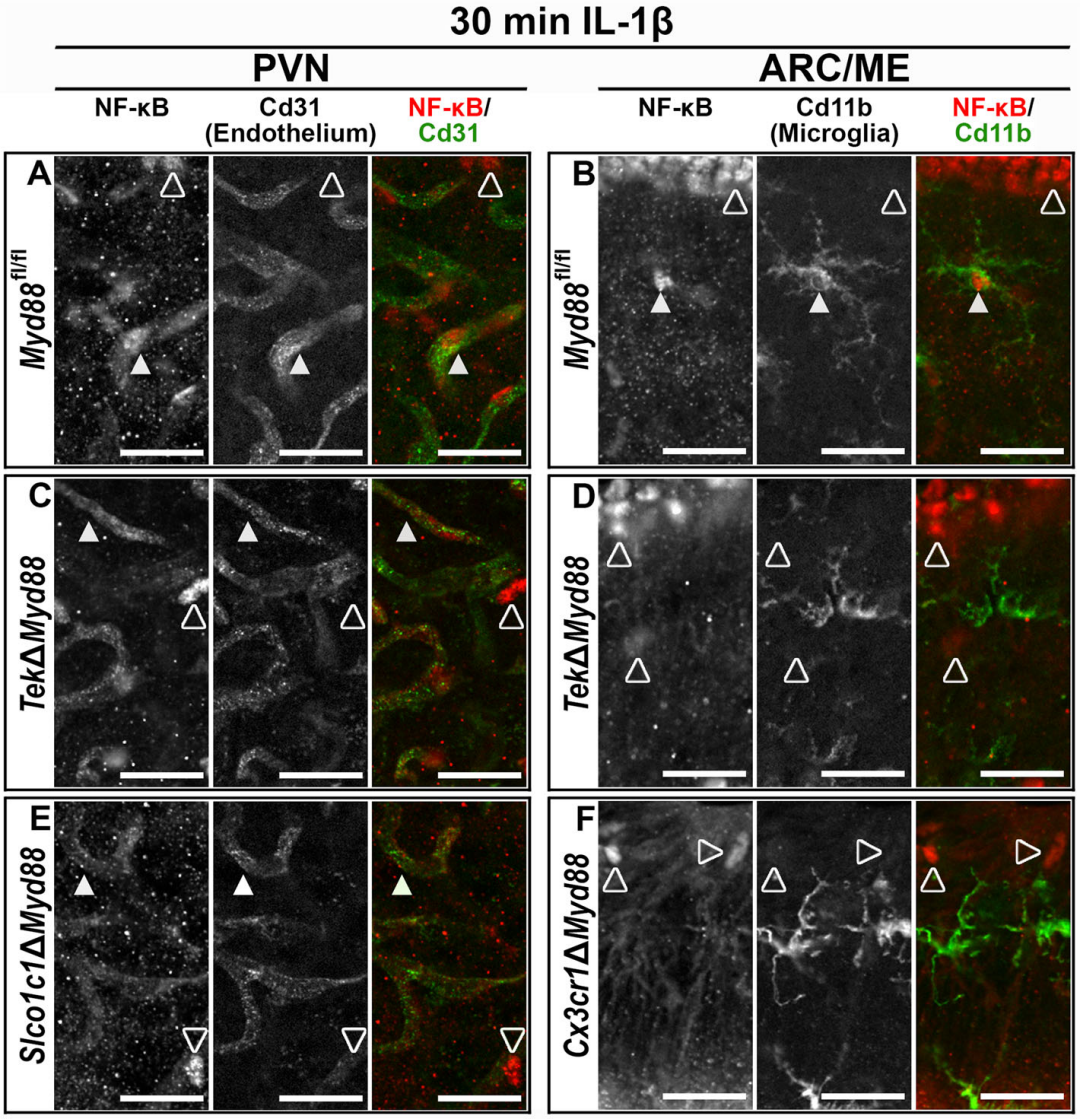
NF-κB immunoreactivity (IR) confirms strain-specific IL-1β signaling disruption. Representative confocal images show that in control animals (*Myd88* ^fl/fl^), IL-1β causes nuclear localization of NF-κB (red) in endothelial cells (Cd31+, green; filled arrowheads in **a** and microglia (Cd11b+, green; filled arrowheads in **b**, *n* = 3). Nuclear IR was also present in cells that did not express co-labeled proteins (open arrowheads in **a**–**f**). *Tek*Δ*Myd88* disrupts IL-1β signaling in endothelium and microglia as NF-κB remains cytoplasmic in blood vessels (filled arrows in **c**) and nuclear IR was absent from microglia (**d**, *n* = 3). The vascular pattern of cytoplasmic NF-κB IR shows that *Slco1c1*Δ*Myd88* disrupts signaling in parenchymal endothelium (filled arrowheads in **e**, *n* = 3). The absence of nuclear NF-κB in microglia of *Cx3cr1*Δ*Myd88* demonstrates microglia signaling disruption (**f**, *n* = 3). Scale bars = 25 μm

To confirm *Myd88* deletion and IL-1β signaling disruption, we examined NF-κB IR in brain sections from Cre− control (*Myd88* ^fl/fl^) and Δ*Myd88* littermates 30 min after 10 ng ICV IL-1β treatment. In *Myd88* ^fl/fl^ animals, IL-1β treatment caused nuclear localization of NF-κB IR in endothelial cells within the PVN (filled arrowheads in Fig. 5a). In contrast, a vascular pattern of cytoplasmic NF-κB IR is evident in the PVN of IL-1β treated *Tek*Δ*Myd88* and *Slco1c1*Δ*Myd88* animals, indicating that Myd88-dependent IL-1β is disrupted in the endothelial cells of these animals (filled arrowheads in Fig. 5c, e). Analysis of endothelial nuclear NF-κB revealed a highly significant effect of genotype (total number of NF-κB+ nuclei surrounded by Cd31 IR: *Myd88* ^fl/fl^ = 92.33 ± 13.68, *Tek*Δ*Myd88 =* 3.333 ± 2.404, and *Slco1c1*Δ*Myd88 =* 0.3333 ± 0.3333, mean ± SEM; genotype *F* (2, 6) = 42.47, *p* = 0.0003, *n* = 3 for all). The number of NF-κB+ endothelial nuclei in both conditional knockouts was significantly different from control animals (*p* = 0.0006 and 0.0005 for *Myd88* ^fl/fl^ vs. *Tek*Δ*Myd88* and *Slco1c1*Δ*Myd88*, respectively), and there was no difference between the *Tek*Δ*Myd88* and *Slco1c1*Δ*Myd88* lines (*p* = 0.9624). Similarly, while many microglia in the ARC/ME of control animals had nuclear NF-κB IR (filled arrowheads in Fig. 5b), almost none were found in either *Tek*Δ*Myd88* or *Cx3cr1*Δ*Myd88* animals (Fig. 5d, f). The effect of genotype on nuclear localization of NF-κB in microglia was highly significant (total number of NF-κB+ nuclei surrounded by Cd11b IR: *Myd88* ^fl/fl^ = 48.67 ± 4.372, *Tek*Δ*Myd88* = 8 ± 6.506, and *Cx3cr1*Δ*Myd88 =* 0.6667 ± 0.3333, mean ± SEM; effect of genotype *F* (3, 8) = 31.42, *p* < 0.0001, *n* = 3 for all). The number of NF-κB+ microglial nuclei in both conditional knockouts was significantly different from control animals (*p* = 0.0004 and 0.0001 for *Myd88* ^fl/fl^ vs. *Tek*Δ*Myd88* and *Cx3cr1*Δ*Myd88*, respectively), and there was no difference between *Tek*Δ*Myd88* and *Cx3cr1*Δ*Myd88* animals (*p* = 0.5935). The presence of nuclear NF-κB IR in ependymal cells and within the parenchyma of the brain confirms that disruption of Myd88-dependent IL-1β signaling is not global in any of the conditional knockouts, but is restricted to the sites of Cre expression (open arrowheads in Fig. 5c–f).

**Fig. 6:**
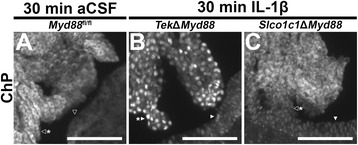
NF-κB immunoreactivity (IR) in the choroid plexus confirms promotor-specific genetic recombination. Representative epifluorescent images of the choroid plexus (ChP) and neighboring ependyma 30 min after treatment confirms genetic recombination of *Myd88* in a pattern consistent with reporter crosses (see Fig. 4a, h). In vehicle-treated (artificial cerebrospinal fluid, aCSF) control animals (*Myd88* ^fl/fl^, *n* = 3; **a**), clear nuclear voids of NF-κB IR are present in the cuboidal cells of the ChP (open arrowhead with asterisk) and only diffuse, cytoplasmic labeling, without concentrated nuclear labeling, is seen in the ependyma (open arrowhead). As with control animals (see Fig. 2k–l and Additional file 3: Figure S3A, B), nuclear NF-κB is evident in both cuboidal (arrowhead with asterisk in **b**) and ependymal cells (arrowhead in **b**) in response to 10 ng intracerebroventricular IL-1β treatment of *Tek*Δ*Myd88* (*n* = 3), which do not express Cre in either cell type. In contrast, while nuclear NF-κB is present in the ependymal cells (arrowhead in **c**) of IL-1β-treated *Slco1c1*Δ*Myd88* animals (*n* = 3), NF-κB IR remains cytoplasmic in ChP cuboidal cells where *Slco1c1*-Cre is expressed (arrowhead with asterisk in **c**; compare with Fig. 4h). Scale bars = 100 μm

Examination of NF-κB IR in the ChP provides further evidence of promotor-specific *Myd88* deletion and confirms that TdT expression corresponds to sites of genetic recombination (Fig. 6). In vehicle-treated control animals, ChP cuboidal cells display clear nuclear voids of NF-κB IR (open arrowhead with asterisk) and NF-κB IR in ependymal cells is diffuse rather than concentrated within the nuclei (open arrowhead; Fig. 6a). As previously demonstrated (see Fig. 2l and Additional file 3: Figure S3B, C), IL-1β treatment reliably causes nuclear localization of NF-κB IR in ChP cuboidal cells and ependymal cells of control animals. Similarly, IL-1β treatment resulted in nuclear NF-κB in ependymal cells in both *Tek*Δ*Myd88* and *Slco1c1*Δ*Myd88* mice (filled arrowheads in Fig. 6b, c). In contrast, IL-1β treatment resulted in nuclear NF-κB in the cuboidal cells of *Tek*Δ*Myd88* (filled arrowhead with asterisk in Fig. 6b) but not *Slco1c1*Δ*Myd88* mice (open arrowhead with asterisk in Fig. 6c). These results corroborate those presented in Fig. 4 and demonstrate that Cre-dependent disruption of IL-1β signaling occurs in the cellular populations identified by reporter expression.

Of the three Cre lines used in this series of experiments, the *Cx3cr1*-CreERT2 line is unique in that it is a knock-in at the endogenous locus rather than a random insertion. This has the advantage of maintaining potential regulatory elements and thus ensuring transgene expression in a biologically relevant distribution but is a distinct disadvantage for these experiments as both *Cx3cr1* and *Myd88* are located on chromosome 9. To generate *Cx3cr1*Δ*Myd88* mice, it was necessary to breed compound heterozygous mice (*Cx3cr1*-Cre^+/−^ *Myd88* ^fl/WT^) until spontaneous genetic recombination was detected. While breeding was ongoing, a line of mice carrying a conditional allele of interleukin 1 receptor 1 (*Il1r1* ^fl/fl^) became available, allowing disruption of IL-1β signaling in microglia independent of Myd88 (*Cx3cr1*Δ*Il1r1*) [29]. NF-κB IR in brain sections from IL-1β-treated control (*Cx3cr1*-CreERT2/YFP^+/−^, *Myd88*/*Il1r1* ^WT/WT^; *Cx3cr1* ^+/WT^), *Cx3cr1*Δ*Myd88*, and *Cx3cr1*Δ*Il1r1* animals confirms that both *Cx3cr1*-CreERT2 knockout lines eliminated the response to IL-1β in almost all microglia (Additional file 6: Figure S6). As with *Cx3cr1*Δ*Myd88*, the number of microglia with NF-κB+ nuclei was significantly different between IL-1β-treated control (*Il1r1* ^fl/fl^) and *Cx3cr1*Δ*Il1r1* mice (total number of NF-κB+ nuclei surrounded by Cd11b IR: *Il1r1* ^fl/fl^ = 58.67 ± 5.925 and *Cx3cr1*Δ*Il1r1 =* 3.333 ± 1.764; *p* = 0.012). These results indicate that *Cx3cr1*-CreERT2 can be used to disrupt IL-1β signaling in microglia using conditional alleles of either *Myd88* or *Il1r1*.

**Fig. 7:**
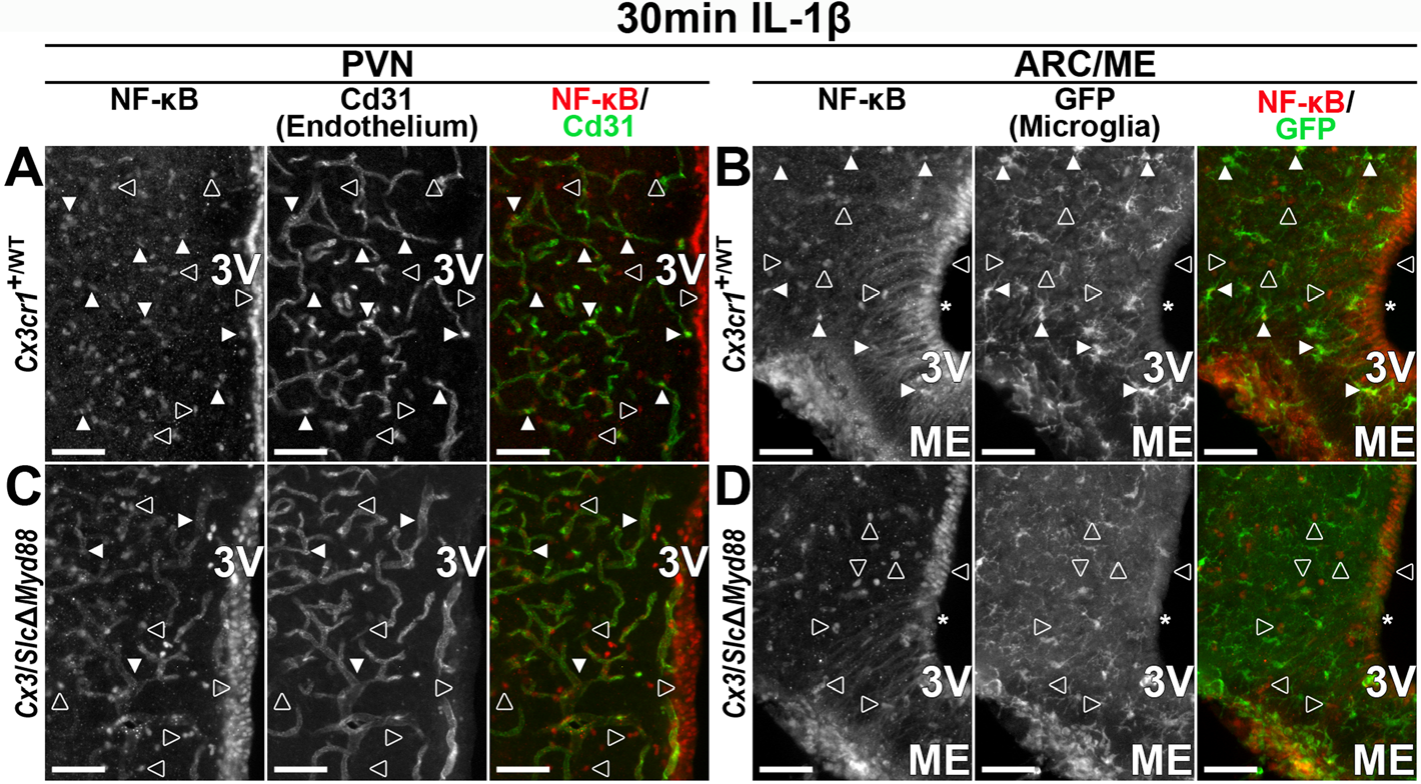
Combined *Cx3/Slc*-CreERT2 eliminates IL-1β signaling in parenchymal endothelium and microglia. Representative epifluorescent images of the paraventricular nucleus (PVN, **a**) and arcuate nucleus/median eminence region (ARC/ME, **b**) showing IL-1β-induced nuclear NF-κB immunoreactivity (IR) in parenchymal endothelium (co-expression with Cd31 indicated by filled arrowheads in **a**) and GFP+ microglia (filled arrowheads in **b**) in *Cx3cr1*-CreERT2+ animals that do not have floxed *Myd88* (*Cx3cr1* ^+/WT^, *n* = 5). Nuclear NF-κB IR is also found in cells that do not express either marker (open arrowheads in **a** and **b**), including ependymal cells lining the third ventricle (3V) and β1-tanycytes (asterisk in **b**). Representative images from an IL-1β-treated combined Cre animal (*Cx3/Slc*Δ*Myd88*, *n* = 3) showing cytoplasmic NF-κB IR in PVN parenchymal endothelium (filled arrowheads in **c**) and an absence of nuclear NF-κB IR in GFP+ microglia (**d**) demonstrating that these cells do not respond to ICV IL-1β in combined Cre animals. Similar to control animals, nuclear NF-κB IR is found in cells that do not express either marker (open arrowheads in **c** and **d**), but with an apparent decrease in β1-tanycytes (asterisk in **d**) where *Slco1c1*-CreERT2 is expressed (see Fig. 4g). Scale bars = 50 μm

Once they were available, we crossed the *Cx3cr1*Δ*Myd88* and *Slco1c1*Δ*Myd88* lines to generate a compound knockout where *Myd88* is deleted in both microglia and all brain vessels except fenestrated capillaries (*Cx3/Slc*Δ*Myd88*). NF-κB IR in brain sections from tamoxifen-treated control (*Cx3cr1* ^+/WT^) and *Cx3/Slc*Δ*Myd88* animals 30 min after ICV IL-1β treatment demonstrates that the compound knockout combines the phenotypes of the individual knockouts, essentially eliminating the IL-β response in parenchymal endothelium (*Slco1c1*Δ*Myd88*) and microglia (*Cx3cr1*Δ*Myd88*; Fig. 7). In brain sections from *Cx3cr1* ^+/WT^ animals, nuclear NF-κB IR was present in parenchymal endothelium within the PVN (filled arrowheads in Fig. 7a) and in microglia and β1 tanycytes in the ARC/ME (filled arrowheads and asterisk in Fig. 7b). In the PVN of *Cx3/Slc*Δ*Myd88* animals, there was a clear vascular pattern of cytoplasmic NF-κB IR with a marked decrease in endothelial nuclear NF-κB, indicating that IL-1β does not cause NF-κB nuclear translocation in parenchymal endothelium (Fig. 7c). The vascular pattern of NF-κB IR seen in IL-1β-treated *Cx3/Slc*Δ*Myd88* animals is comparable to that seen in vehicle-treated control animals and in IL-1β-treated *Slco1c1*Δ*Myd88* animals (see Figs. 2a and 5e). As in IL-1β-treated *Cx3cr1*Δ*Myd88* animals (see Fig. 5f), microglia in the ARC/ME of IL-β-treated *Cx3/Slc*Δ*Myd88* animals lacked nuclear labeling (Fig. 7d). Similarly, β1 tanycytes in the ARC/ME of IL-1β-treated *Cx3/Slc*Δ*Myd88* animals had an apparent decrease in nuclear NF-κB (asterisk in Fig. 7d), indicating signaling disruption in this *Slco1c1*-CreERT2 expressing cell type (see Fig. 4g). The presence of nuclear NF-κB in other cell types in *Cx3/Slc*Δ*Myd88* animals indicates that signaling disruption is not global (open arrowheads in Fig. 7c, d). These results show that the *Cx3/Slc*Δ*Myd88* line can be used to eliminate IL-1β signaling in both parenchymal endothelium and microglia.

### Disruption of IL-1β signaling in endothelium and microglia eliminates the sickness response

**Fig. 8:**
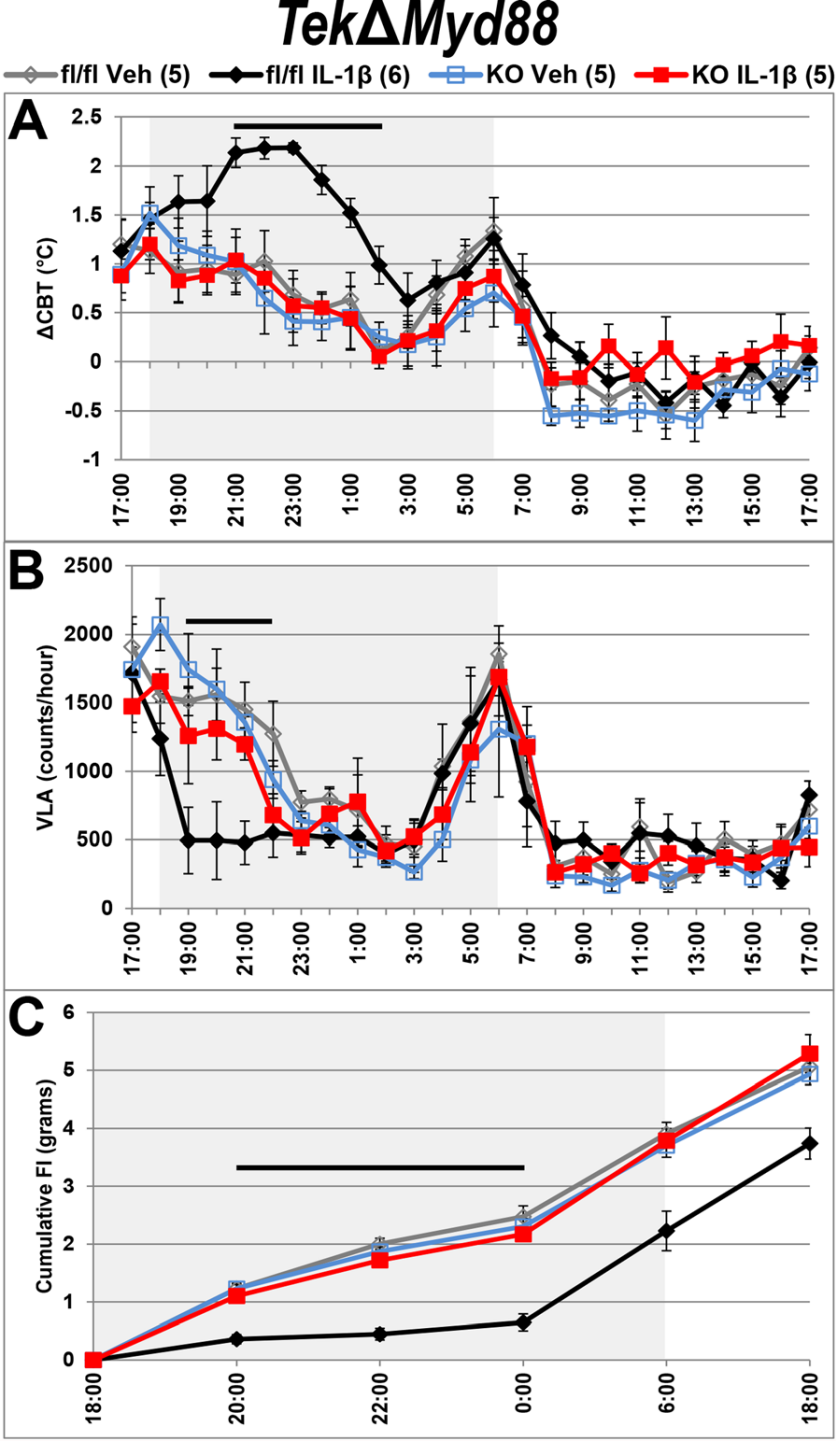
*Tek*Δ*Myd88*-mediated disruption of IL-1β signaling in all endothelium and microglia eliminates sickness response. Twenty-four-hour profiles of telemetric and feeding data shows the stereotypical IL-1β-induced elevation in core body temperature (ΔCBT, **a**) and decrease in voluntary locomotor activity (VLA, **b**) and food intake (FI, **c**) in *Myd88* ^fl/fl^ (fl/fl) but not *Tek*Δ*Myd88* (KO) mice. IL-1β-treated control ΔCBT, VLA and FI were significantly different from vehicle (Veh)-treated values for several hours following treatment (*p* < 0.05 for times below black bar above traces in **a**–**c**). IL-1β-treated KO animals were not different from Veh-treated animals at any time. Gray boxes show dark phase, when mice are most active. All values shown are mean ± SEM for group sizes listed in the legend above **a**

We examined the effects of ICV IL-1β on *Myd88* ^fl/fl^ (fl/fl) and *Tek*Δ*Myd88* (KO) littermates by monitoring core body temperature (CBT), voluntary locomotor activity (VLA), food intake (FI), and body weight (BW) for 24 h after treatment. In agreement with previous reports, IL-1β treatment of *Myd88* ^fl/fl^ animals resulted in stereotypical patterns of significantly increased core body temperature (ΔCBT) and decreased VLA and FI compared to vehicle (Veh)-treated animals (Fig. 8). These changes lasted for several hours, with fever peaking 4 to 5 h after treatment. In contrast, all measured parameters of IL-1β-treated *Tek*Δ*Myd88* animals were indistinguishable from those of their vehicle-treated counterparts.

**Fig. 9:**
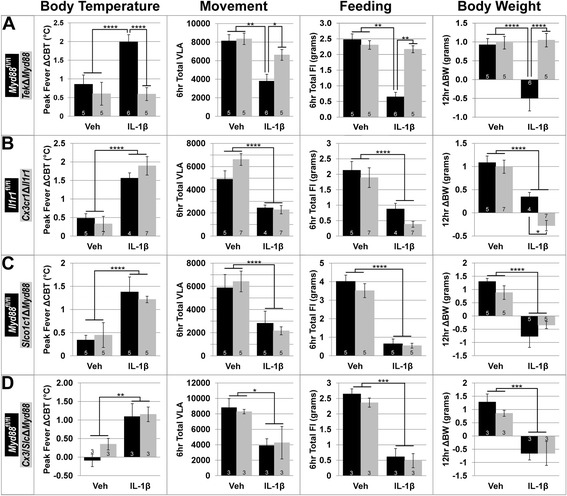
IL-1β-induced sickness responses are only eliminated when signaling is disrupted in all endothelium and microglia. *Tek*Δ*Myd88*-mediated disruption of IL-1β signaling in all endothelium and microglia eliminates the increase in core body temperature (ΔCBT) and decrease in voluntary locomotor activity (VLA), food intake (FI), and body weight (ΔBW) associated with IL-1β treatment of *Myd88* ^fl/fl^ mice (**a**). Targeted disruption of IL-1β signaling in microglia alone (*Cx3cr1*Δ*Il1r1*, **b**), parenchymal endothelium (*Slco1c1*Δ*Myd88*, **c**) or both parenchymal endothelium and microglia (*Cx3/Slc*Δ*Myd88*, **d**) was insufficient to alter the sickness response. IL-1β treatment is the only factor that influences ΔCBT, VLA, FI, and ΔBW for all genotypes except *Tek*Δ*Myd88*. All values shown are mean ± SEM for group sizes listed in graph bars. *p < 0.05; **p < 0.01; ***p < 0.001; ****p < 0.0001

To quantify these differences, we analyzed the average ΔCBT at the peak of fever (10 pm–12 am), total VLA counts, and FI for the first 6 h after treatment (6 pm–12 am) and change in body weight (ΔBW) 12 h after treatment (6 pm–6 am; Fig. 9a). In each case, two-way ANOVA revealed a significant effect of treatment and genotype on ΔCBT (treatment *F* (1, 17) = 30.22, *p* < 0.0001; genotype *F* (1, 17) = 64.98, *p* < 0.0001), VLA (treatment *F* (1, 17) = 20.47, *p* = 0.0003; genotype *F* (1, 17) = 5.178, *p* = 0.036), FI (treatment *F* (1, 17) = 43.53, *p* < 0.0001; genotype *F* (1, 17) = 20.35, *p* = 0.0003), and ΔBW (treatment *F* (1, 17) = 7.552, *p* = 0.014; genotype *F* (1, 17) = 10.77, *p* = 0.0044). For all but VLA, there was also a significant interaction of treatment by genotype (ΔCBT: *F* (1, 17) = 31.08, *p* < 0.0001; VLA: *F* (1, 17) = 3.746, *p* = 0.07; FI: *F* (1, 17) = 32.75, *p* < 0.0001; ΔBW: *F* (1, 17) = 9.213, *p* = 0.0075). Post hoc analysis revealed that all differences were due to the effects of IL-1β on *Myd88* ^fl/fl^ animals, as only this group was significantly different from all other groups for ΔCBT (mean ± SEM: IL-1β-*Myd88* ^fl/fl^ = 1.99 ± 0.19 °C vs. IL-1β-*Tek*Δ*Myd88* = 0.59 ± 0.17 °C, Veh-*Myd88* ^fl/fl^ = 0.86 ± 0.24 °C, Veh-*Tek*Δ*Myd88* = 0.60 ± 0.31 °C; *p* < 0.0001 for all), VLA (mean ± SEM: IL-1β-*Myd88* ^fl/fl^ = 3792 ± 733 vs. IL-1β-*Tek*Δ*Myd88* = 6616 ± 583, Veh-*Myd88* ^fl/fl^ = 8124 ± 687, Veh-*Tek*Δ*Myd88* = 8352 ± 614; *p* = 0.033, 0.0011, 0.0007 respectively), FI (mean ± SEM: IL-1β-*Myd88* ^fl/fl^ = 0.65 ± 0.15 g vs. IL-1β-*Tek*Δ*Myd88* = 2.17 ± 0.11 g, Veh-*Myd88* ^fl/fl^ = 2.48 ± 0.18 g, Veh-*Tek*Δ*Myd88* = 2.3 ± 0.14 g; *p* < 0.0001 for all), and ΔBW (mean ± SEM: IL-1β-*Myd88* ^fl/fl^ = − 0.49 ± 0.34 g vs. IL-1β-*Tek*Δ*Myd88* = 1.05 ± 0.18 g, Veh-*Myd88* ^fl/fl^ = 0.92 ± 0.17 g, Veh-*Tek*Δ*Myd88* = 0.98 ± 0.17 g; *p* = 0.0014, 0.0032, 0.0022 respectively). IL-1β-treated *Tek*Δ*Myd88* animals were not different from vehicle-treated animals of either genotype for any parameter measured, demonstrating that IL-1β-induced sickness responses are dependent upon Myd88 expression in endothelium and/or microglia.

### Disruption of IL-1β signaling exclusively in microglia does not affect the sickness response

Because *Tek*-Cre is expressed in both endothelium and microglia, it is impossible to determine the individual contribution of either cell type alone using the *Tek*Δ*Myd88* line. We examined the role of microglia independent of endothelium using the microglia-specific *Cx3cr1*-CreERT2. In both *Cx3cr1*Δ*Il1r1* and *Cx3cr1*Δ*Myd88*, the response to ICV IL-1β in experimental animals (KO) and their control littermates (fl/fl) was the same. IL-1β treatment of *Cx3cr1*Δ*Il1r1* and *Il1r1* ^fl/fl^ mice caused stereotypical fever and reduction in VLA and FI for several hours after treatment (Additional file 7: Figure S7A–C). Two-way ANOVA revealed a highly significant effect of treatment (Fig. 9b), no effect of genotype, and no interaction of treatment by genotype for ΔCBT (0.48 ± 0.12 °C and 0.33 ± 0.2 °C vs. 1.56 ± 0.14 °C and 1.89 ± 0.25 °C; mean ± SEM for *Il1r1* ^fl/fl^ and *Cx3cr1*Δ*Il1r1* Veh vs. IL-1β, respectively; treatment *F* (1, 19) = 37.13, *p* < 0.0001), VLA (4903 ± 732 and 6611 ± 508 vs. 2435 ± 249 and 2276 ± 359; mean ± SEM for *Il1r1* ^fl/fl^ and *Cx3cr1*Δ*Il1r1* Veh vs. IL-1β, respectively; treatment *F* (1, 18) = 41.92, *p* < 0.0001), and FI (2.13 ± 0.28 g and 1.89 ± 0.32 g vs. 0.88 ± 0.18 g and 0.38 ± 0.1 g; mean ± SEM for *Il1r1* ^fl/fl^ and *Cx3cr1*Δ*Il1r1* Veh vs. IL-1β, respectively; treatment *F* (1, 19) = 29.77, *p* < 0.0001). Analysis of body weight data revealed a highly significant effect of treatment (*F* (1, 19) = 55.4, *p* < 0.0001), a significant effect of genotype (*F* (1, 19) = 6.927, *p* = 0.0164), and no interaction of treatment by genotype for ΔBW (1.08 ± 0.14 g and 0.99 ± 0.14 g vs. 0.35 ± 0.1 g and − 0.28 ± 0.11 g; mean ± SEM for *Il1r1* ^fl/fl^ and *Cx3cr1*Δ*Il1r1* Veh vs. IL-1β, respectively). The effect of genotype on ΔBW reflects the fact that IL-1β-treated *Il1r1* ^fl/fl^ animals did not lose as much weight as *Cx3cr1*Δ*Il1r1* animals.

Analysis of the effects of ICV IL-1β on *Cx3cr1*Δ*Myd88* animals revealed a significant effect of treatment, no effect of genotype, and no interaction of treatment by genotype for both FI (4.83 ± 0.56 g and 5.3 ± 0.23 g vs. 2.57 ± 0.53 g and 3.25 ± 0.71 g; mean ± SEM for *Myd88* ^fl/fl^ and *Cx3cr1*Δ*Myd88* Veh vs. IL-1β, respectively; treatment *F* (1, 8) = 16.32, *p* = 0.0037) and ΔBW (0.5 ± 0.2 g and 0.84 ± 0.12 g vs. − 0.86 ± 0.25 g and − 0.7 ± 0.66 g; mean ± SEM for *Myd88* ^fl/fl^ and *Cx3cr1*Δ*Myd88* Veh vs. IL-1β, respectively; treatment *F* (1, 8) = 15.28, *p* = 0.0045; Additional file 8: Figure S8). Together, these results clearly demonstrate that disruption of IL-1β signaling in microglia alone does not alter the sickness response to ICV IL-1β.

### Deletion of Myd88 from brain parenchymal endothelium does not alter the sickness response

Having eliminated microglia as the sole causative agent of the IL-1β-induced sickness response, we queried the contribution of blood vessels independent of microglia using *Slco1c1*-CreERT2 mice. ICV IL-1β caused increased ΔCBT and decreased VLA and FI for both groups relative to their vehicle-treated counterparts for several hours after treatment (Additional file 7: Figure S7D–F). Similar to the *Cx3cr1*-CreERT2 results, we found no differences between *Slco1c1*Δ*Myd88* and *Myd88* ^fl/fl^ animals (Fig. 9c). There was a highly significant effect of treatment with no effect of genotype or interaction of treatment by genotype for ΔCBT (0.34 ± 0.10 °C and 0.45 ± 0.27 °C vs. 1.38 ± 0.32 °C and 1.22 ± 0.07 °C; mean ± SEM for *Myd88* ^fl/fl^ and *Slco1c1*Δ*Myd88* Veh vs. IL-1β, respectively; treatment *F* (1, 16) = 17, *p* = 0.0008), VLA (8191 ± 1146 and 8388 ± 893 vs. 2282 ± 1042 and 2235 ± 348; mean ± SEM for *Myd88* ^fl/fl^ and *Slco1c1*Δ*Myd88* Veh vs. IL-1β, respectively; treatment *F* (1, 16) = 25.05, *p* < 0.0001), FI (4.02 ± 0.34 g and 3.52 ± 0.38 g vs. 0.65 ± 0.26 g and 0.54 ± 0.13 g; mean ± SEM for *Myd88* ^fl/fl^ and *Slco1c1*Δ*Myd88* Veh vs. IL-1β, respectively; treatment *F* (1, 16) = 38.11, *p* < 0.0001), and ΔBW (1.3 ± 0.12 g and 0.88 ± 0.26 g vs. − 0.77 ± 0.42 g and − 0.34 ± 0.14 g; mean ± SEM for *Myd88* ^fl/fl^ and *Slco1c1*Δ*Myd88* Veh vs. IL-1β, respectively; treatment *F* (1, 16) = 39.37, *p* < 0.0001). These results demonstrate that elimination of Myd88-dependent signaling in brain parenchymal endothelium, but not in microglia or fenestrated capillaries, is insufficient to alter the sickness response to IL-1β.

### Deletion of Myd88 from brain parenchymal endothelium and microglia does not alter the sickness response

Taken together, the previous results suggest two possible conclusions: (1) Myd88 expression in either blood vessels or microglia alone is sufficient to maintain the IL-1β-induced sickness response, and it is only when Myd88-dependent IL-1β signaling is disrupted in both cell types simultaneously that the sickness response is eliminated; or (2) Myd88 expression in CNS vessels that express *Tek*-Cre but not *Slco1c1*-CreERT2—the fenestrated capillaries of CVOs—is sufficient to maintain the IL-1β-induced sickness response. To address this issue, we used the *Cx3/Slc*Δ*Myd88* compound knockout to determine whether elimination of IL-1β signaling in both parenchymal endothelium and microglia is sufficient to alter the sickness response.

In response to IL-1β, both *Myd88* ^fl/fl^ and *Cx3/Slc*Δ*Myd88* mice exhibited increased ΔCBT and decreased VLA and FI for several hours after treatment (Additional file 7: Figure S7G–I). As with the individual knockouts (*Cx3cr1*Δ*Il1r1*, *Cx3cr1*Δ*Myd88*, and *Slco1c1*Δ*Myd88*) and control animals, IL-1β treatment of *Cx3/Slc*Δ*Myd88* animals resulted in the full set of sickness responses (Fig. 9d). There was a significant effect of treatment, no effect of genotype, and no interaction of treatment by genotype for ΔCBT (− 0.09 ± 0.17 °C and 0.35 ± 0.16 °C vs. 1.09 ± 0.35 °C and 1.15 ± 0.2 °C; mean ± SEM, for *Myd88* ^fl/fl^ and *Cx3/Slc*Δ*Myd88* Veh vs. IL-1β, respectively; treatment *F* (1, 8) = 18.07, *p* = 0.0028), VLA (8328 ± 1183 and 8219 ± 521 vs. 4111 ± 1084 and 4311 ± 2078; mean ± SEM, for *Myd88* ^fl/fl^ and *Cx3/Slc*Δ*Myd88* Veh vs. IL-1β, respectively; treatment *F* (1, 8) = 9.221, *p* = 0.016), FI (2.65 ± 0.17 g and 2.37 ± 0.15 g vs. 0.61 ± 0.27 g and 0.48 ± 0.23 g; mean ± SEM, for *Myd88* ^fl/fl^ and *Cx3/Slc*Δ*Myd88* Veh vs. IL-1β, respectively; treatment *F* (1, 8) = 89.06, *p* < 0.0001), and ΔBW (1.28 ± 0.31 g and 0.86 ± 0.21 vs. − 0.66 ± 0.25 g and − 0.66 ± 0.51; mean ± SEM, for *Myd88* ^fl/fl^ and *Cx3/Slc*Δ*Myd88* Veh vs. IL-1β, respectively; treatment *F* (1, 8) = 26.09, *p* = 0.0009). These results show that disruption of Myd88-dependent signaling in parenchymal endothelium and microglia, but not in fenestrated capillaries, is insufficient to eliminate the sickness response.

## Discussion

One of the enduring questions in the field of inflammation research is the precise mechanism and central anatomical location where cytokines produced within the CNS act to generate sickness responses. To address this question, we first examined the pattern of NF-κB IR after central IL-1β administration. For all experiments in this study, we used a dose of IL-1β (10 ng) that has been previously shown to cause sickness responses without elevating circulating IL-1β levels, thereby allowing examination of actions and effects of IL-1β that are confined to the brain [10]. In agreement with previous studies, we found that IL-1β treatment results in a change from a diffuse, cytoplasmic distribution to a punctate, nuclear pattern, indicating nuclear translocation of NF-κB in a time- and region-dependent manner [24, 52]. The fact that this change is absent in IL-1β-treated *Myd88*KO animals, which do not exhibit sickness responses when given IL-1β, demonstrates that NF-κB nuclear translocation is dependent on Myd88 [12]. It also validates NF-κB IHC as a tool for both visualizing the sites of IL-1β action and for verifying Cre-mediated signaling disruption.

We found that vascular endothelium, choroid plexus, ependyma, astrocytes, and microglia all demonstrate nuclear translocation of NF-κB following ICV IL-1β administration. Although the concentration of NF-κB+ nuclei was highest around CVOs, we observed nuclear translocation throughout the brain. Such a broad response complicates the task of determining which components are critical for the physiologic and behavioral sickness responses. For example, microglia undergo activation in response to inflammatory stimuli, altering their transcription profiles and appearance [53–55]. Components of the BBB, including microvascular endothelial cells and ependymal cells, also undergo cytoarchitectural modifications in inflammatory settings, potentially altering the diffusion barriers between the blood, CSF, and brain [56, 57]. Both of these lead to profound changes within the CNS, but this does not prove that they are essential for generating sickness responses.

Although multiple different cell types demonstrate nuclear localization of NF-κB in response to IL-1β, there are some apparent differences in the intensity of immunohistochemical labeling of NF-κB. For example, in sections from vehicle-treated animals (unstimulated state), ChP cuboidal epithelial cells demonstrate cytoplasmic labeling, with obvious nuclear voids in immunoreactivity. Similarly, cytoplasmic labeling of unstimulated endothelial cells is visible in a branching, vascular pattern, though without such obvious absences of nuclear immunoreactivity. In other cell types, such as microglia, astrocytes, and ependymal cells, cytoplasmic labeling of NF-κB is not as obvious in the unstimulated state. There are also cell-type-specific differences in NF-κB IR in sections from IL-1β-treated animals (stimulated state). For example, while both cuboidal and ependymal cells clearly demonstrate IL-1β-induced nuclear localization of NF-κB (see Additional file 3: Figure S3B, C), the apparent intensity of immunoreactivity is much higher in stimulated cuboidal cells than in ependymal cells (see Fig. 6). While it should be noted that the immunohistochemical method used to detect NF-κB in these studies is not quantitative, differences in immunoreactivity could be informative. Such differences could be due to a number of factors including differences in the amount of NF-κB expressed by the different cell types, differences in cellular morphology that manifests as a difference in optical detection, or cell-type-specific differences in membrane permeability allowing for differential antibody access. Whether the apparent visual differences in immunoreactivity are biologically meaningful is not known.

To determine which IL-1β responsive cells are necessary for the resultant fever, lethargy, anorexia, and loss of body weight, we systematically eliminated Myd88-dependent signaling from identified targets. Previously, we found that deletion of *Myd88* in neurons and astrocytes (utilizing *Nes*-Cre mice) did not affect the behavioral response to IL-1β [12]. This finding was surprising considering that a behavioral response to a stimulus requires neuronal involvement. Indeed, we and others presented evidence that ICV IL-1β induces activation of restricted populations of neurons involved in regulating appetite, body temperature, metabolic homeostasis, and hedonistic behaviors [24, 52, 58, 59]. An early study of IL-1β in rats found that neurons activated in the PVN and elsewhere do not express Il1r1, indicating an indirect mechanism of stimulation [60]. The current result that PVN neurons do not display nuclear localization of NF-κB following ICV IL-1β is further evidence that alterations in neuronal activity are not exclusively due to direct action of IL-1β but instead require an intermediate signal from a different cell type.

Prostaglandin E2 (PGE2) is one example of an intermediate signaling molecule that plays a role in the sickness response. Long recognized as an important endogenous pyrogen, PGE2 might also play a role in generating other sickness responses [61]. PGE2 is produced in the brain by endothelial cells and microglia in response to inflammatory stimuli [62–64]. Wilhelms et al. reported that deletion of PGE2 synthesizing enzymes from vascular endothelium using the *Slco1c1*-CreERT2 line reduces the febrile response to peripheral LPS and IL-1β without affecting changes in locomotor activity [18]. In a similar study, Ridder et al. report that endothelial deletion of *Tak1*, a component of the IL-1β signaling cascade, using the *Slco1c1*-CreERT2 line reduced the febrile and lethargic responses to intravenous IL-1β without affecting anorexia, weight loss, or corticosterone production [17]. The loss of IL-1β-induced PGE2 production by endothelium and/or microglia could explain our result that the febrile response is eliminated only in the *Tek*Δ*Myd88* line; only *Tek*-Cre causes recombination in microglia and all endothelium, including fenestrated capillaries, thus affecting all sources of PGE2.

Alternatively, it is possible that a specific cellular or regional source of PGE2 is responsible for fever. PGE2 causes fever by directly activating neurons in the thermoregulatory median preoptic nucleus (MnPOA), a structure adjacent to the OVLT [65]. Previous reports demonstrated inflammatory stimulus-induced expression of the prostaglandin-synthesizing enzyme cyclooxygenase-2 (COX-2) in the OVLT, one of the CVOs where we observed the highest density of IL-1β-induced nuclear NF-κB [15, 62]. This raises the possibility that PGE2 produced specifically within the OVLT acts on the nearby MnPOA neurons to generate fever, while other cellular/regional sources might contribute to different aspects of the sickness response. For example, PGE2 can activate neurons in the PVN that control the hypothalamic-pituitary-adrenal (HPA) axis, which in turn drives muscle catabolism, a hallmark of sickness-induced wasting [10, 64, 66].

In contrast, other studies demonstrate that while genetic disruption of PGE2 production eliminates the febrile response to IL-1β, it does so without affecting inflammation-induced depression of locomotor activity [67]. Similarly, Fritz et al. found that *Slco1c1*Δ*Myd88* mice did not demonstrate IL-1β-induced place aversion, but had a normal anorexia response, further supporting our conclusion that anorexia is not dependent on IL-1β signaling in parenchymal endothelium [68]. This same group showed that LPS- and IL-1β-induced conditioned place avoidance requires Myd88-dependent PGE2 production in endothelial cells but not microglia. This result is in agreement with the current finding that genetic disruption of IL-1β signaling in microglia did not affect any of the sickness responses that we measured. Collectively, these results demonstrate that sickness responses can be disrupted individually, rather than collectively, and implicate a subset of vascular endothelium as a critical relay site in the initiation of sickness responses to IL-1β.

The fenestrated capillaries found in CVOs and ChP represent one such category of specialized vasculature. Our results using *Tek*Δ*Myd88* and *Slco1c1*Δ*Myd88* show that fenestrated capillaries are capable of transducing inflammatory signals into sickness responses. Crosses with the Rosa26-flox-stop-TdTomato reporter strain revealed that although there were some differences in reporter expression between the two strains—notably *Tek*- but not *Slco1c1*-Cre expression in microglia and fenestrated capillaries—each drives recombination in all parenchymal brain endothelium. Since both strains eliminate nearly all parenchymal endothelial IL-1β signaling, any observed differences between the two strains are due to the non-overlapping areas of Cre expression; in this case, *Tek*-driven *Myd88* deletion in microglia and/or fenestrated capillaries is responsible for the absence of sickness response in *Tek*Δ*Myd88* animals. Considering that *Cx3cr1*-mediated disruption of microglial IL-1β signaling—even when combined with *Slco1c1*-mediated vascular disruption—failed to affect the sickness response, the most logical conclusion is that the differences between the two vascular Cre strains is due to Myd88 expression in fenestrated capillaries alone. It is currently not known why *Slco1c1-CreERT2* is not expressed in fenestrated capillaries. Because *Tek* is necessary for vascular development and growth, it is not surprising that expression of the *Tek*-Cre transgene is ubiquitous in all endothelial cells [46]. One possible explanation for the absence of *Slco1c1*-CreERT2 activity in fenestrated capillaries is that expression of Slco1c1 is not necessary in these vessels. Thyroid hormone (TH) is important for a number of metabolic processes in nearly every cell of the body, and thus, expression of the TH transporter Slco1c1 by brain parenchymal vasculature ensures that cells within the CNS are exposed to circulating TH. Fenestrated capillaries, due to their altered BBB, should allow TH to more freely diffuse to those areas immediately surrounding them, making Slco1c1 expression unnecessary. It has been previously reported that *Slco1c1-CreERT2* is not expressed in vasculature outside of the CNS, supporting the hypothesis that BBB development induces its expression specifically within the CNS [17]. This hypothesis could be tested by examining endothelial cells cultured in the presence or absence of BBB-inducing astrocyte co-cultures.

It should be noted that we observed some residual NF-κB nuclear localization in the affected cell types in each of the Cre strains used in this study. It is possible that some of what appears to be nuclear localization of NF-κB in the endothelial cells of IL-1β-treated *Tek*- and *Slco1c1*Δ*Myd88* animals is in fact contained within different cell types that are intimately associated with BBB-isolated blood vessels (such as pericytes or perivascular macrophages). It is also possible that what appears to be nuclear localization is actually cytoplasmic labeling seen in cross section due to the three-dimensional branching of blood vessels. Alternatively, it is possible that nuclear NF-κB seen in the various knockouts is not evidence of a direct response to IL-1β. Some nuclear NF-κB was found in vehicle-treated animals, suggesting that even in the absence of exogenous IL-1β, there is some level of NF-κB activity. Finally, it is possible that differences in the genetic tools used in these experiments result in complete genetic recombination in endothelial cells and that each of the genetic lines, including the tamoxifen-inducible strains, which result in equivalent reductions in detectible nuclear NF-κB indicates similar recombination efficiency; however, the possibility of reduced efficiency resulting in incomplete recombination cannot be completely discounted without further experimentation.

It is possible that Myd88 expression in any endothelial cells, as opposed to specifically in fenestrated capillaries, is sufficient to maintain the sickness response. IL-1β-induced production of diffusible signals such as PGE2 could allow for widespread neuronal activation from any responsive population of endothelium. On the other hand, multiple lines of evidence implicating CVOs in the sickness response make fenestrated capillary-specific signaling a more credible possibility. First, Takahashi et al. found that electrolytic lesion of the SFO reduced the febrile response to intravenous LPS, demonstrating that the SFO is critical in the transduction of circulating signals into physiologic responses [34]. Subsequent studies showing that production of inflammatory cytokines, including IL-1β, is restricted to CVOs demonstrate that these structures are uniquely capable of producing this key inflammatory amplification step [10, 22, 23]. Finally, a recent study examining the CNS effects of peripheral LPS administration produced results remarkably similar to our own. Nakano et al. showed that IP LPS caused nuclear localization of signal transducer and activator of transcription 3 (Stat3) specifically within the CVOs [69]. Taken together, these studies show that the CVOs are an exclusive niche where peripheral immune signals interact with elements of the CNS to generate the sickness response.

## Conclusions

The unique anatomy and physiology of fenestrated capillaries suggests that they are a targetable signaling node for treatments designed to prevent or reverse sickness responses. Here, we demonstrate that disruption of IL-1β signaling in microglia or parenchymal endothelium does not affect IL-1β-induced sickness responses, and it is only when signaling is disrupted in all endothelium, including fenestrated capillaries, that these responses are eliminated. Future studies will be dedicated to determining how these CNS vessels transduce the IL-1β signal into a neuronal response. Unfortunately, there is currently no genetic model that would allow for disruption of IL-1β signaling exclusively in fenestrated capillaries; final confirmation of this pathway will require development of this research tool. This would also allow for identification of the secondary signaling molecule(s) that directly stimulate neuronal circuits responsible for generating the sickness response.

## Additional files

Additional file 1: Figure S1. Vascular heterogeneity in the mouse brain. (A-D) Cd31 IR in epifluorescent digital montages of representative brain sections from animals used for various experiments at bregma (A), bregma − 0.5 (B; asterisk shows the location where a cannula was placed to give access to the lateral ventricle), − 1.0 (C) and − 1.5 mm (D) according to the mouse brain atlas by Paxinos and Franklin [70]. Boxes indicate the five regions of interest shown in Fig. 1. Increased vascular density is evident in the organum vasculosum lamina terminalis (OVLT, 1), subfornical organ (SFO, 2), choroid plexus (ChP, 3), paraventricular nucleus (PVN, 4), and arcuate nucleus/median eminence (ARC/ME, 5). Scale bar = 1 mm. (TIFF 6352 kb)

Additional file 2: Figure S2. IL-1β-induced nuclear localization of NF-κB requires Myd88. Representative epifluorescent images of the effects of central IL-1β. Omission of primary antibody (A) demonstrates that the vascular pattern of cytoplasmic immunoreactivity (IR, arrows) observed in vehicle (aCSF)-treated animals (B, *n* = 3) is specific to the NF-κB antibody. At the level of the arcuate nucleus/median eminence (ARC/ME), nuclear NF-κB IR is evident by 15 min (C, *n* = 4) and peaks around 30 min (D, *n* = 8) after ICV IL-1β treatment. Ependymal cells lining the third ventricle (cuboidal nuclei, arrowheads), tanycytes (columnar nuclei, asterisk), and endothelial cells (arrow in D) demonstrate nuclear NF-κB IR. As with vehicle treated animals, NF-κB IR remained cytoplasmic in IL-1β-treated *Myd88*KO (E, arrows, *n* = 3) animals at all times examined. 3V = third ventricle, ME = median eminence. Scale bars = 100 μm. (TIFF 3612 kb)

Additional file 3: Figure S3. DAPI labeling confirms IL-1β-induced NF-κB nuclear localization. Representative epifluorescent images of NF-κB immunoreactivity (IR; left column) and DAPI labeling of nuclear DNA (middle column) 30 min after treatment demonstrate the change in cellular localization caused by 10 ng intracerebroventricular (ICV) IL-1β. This effect is most obvious in the cuboidal cells of the choroid plexus (ChP), where NF-κB IR is found predominantly in the cytoplasm in sections from vehicle-treated animals (artificial cerebrospinal fluid, aCSF; open arrowhead in A) and concentrated in the nucleus of IL-1β-treated animals (arrowhead in B). Like the ChP, the ependymal cells (C) that form the barrier between the CSF and the brain consistently exhibit NF-κB nuclear localization, serving as an indicator that the animal has been exposed to IL-1β. While there are cells in all tissues, there that do not respond to IL-1β (DAPI-labeled blue nuclei that do not co-label with red NF-κB; right column), puncta of concentrated NF-κB IR overlap DAPI, indicating nuclear localization (filled arrowheads). This is true for all regions of the brain, including the organum vasculosum lamina terminalis (OVLT; D), the subfornical organ (SFO; E), the paraventricular nucleus (PVN; F), and median eminence (ME; G). The high cellular density of the brain, particularly within the OVLT, SFO, and PVN, makes it difficult to distinguish between different cells. Scale bars = 25 μm. (TIFF 2487 kb)

Additional file 4: Figure S4. Neurons in the PVN do not exhibit IL-1β-induced nuclear NF-κB. Representative epifluorescent images show that IL-1β causes nuclear localization of NF-κB (green) within the paraventricular nucleus (PVN; A, arrowheads). Despite a high density of neuronal nuclei (NeuN, red; B, arrows), there was no evidence of co-localization (C, *n* = 3). Scale bars = 50 μm. (TIFF 7122 kb)

Additional file 5: Figure S5. Both *Tek*-Cre and *Slco1c1*-CreERT2 drive recombination in parenchymal endothelium. Representative epifluorescent images demonstrate that TdTomato (TdT, red) expression was present in all parenchymal endothelium (Cd31+, green) in both *Tek-*TdT (A-D, *n* = 4) and *Slco1c1*-TdT (E-H, *n* = 4) animals in all brain regions examined. Expression outside of blood vessels (arrowheads) was also present in both lines in all regions. Scale bars = 50 μm. (TIFF 4799 kb)

Additional file 6: Figure S6. *Cx3cr1*-CreERT2 causes genetic recombination exclusively in microglia. Representative epifluorescent images of NF-κB immunoreactivity (IR) 30 min after ICV IL-1β demonstrate *Cx3cr1*-CreERT2-mediated disruption of signaling in microglia when either the interleukin-1 receptor (*Il1r1*) or *Myd88* is deleted. The *Cx3cr1*-CreERT2 transgene contains a sequence coding for YFP that allows for visualization of microglia using an anti-GFP antibody. In Cre + animals that do not have floxed *Il1r1* or *Myd88* (*Cx3cr1* ^+/WT^, *n* = 5), nuclear NF-κB is found in GFP+ microglia in the arcuate nucleus/median eminence (ARC/ME; co-expression denoted by filled arrowheads in A) and in cells that do not express GFP, including ependymal cells lining the third ventricle (3V; open arrowheads). In Cre + animals that are homozygous for floxed alleles of *Il1r1* (*Cx3cr1*Δ*Il1r1*, B, *n* = 4) or *Myd88* (*Cx3cr1*Δ*Myd88*, C, *n* = 3) nuclear NF-κB IR is mostly absent from GFP+ microglia. Nuclear NF-κB IR in cells that do not express GFP (open arrowheads in B and C) demonstrates that IL-1β signaling is not disrupted globally. Scale bars = 50 μm. (TIFF 1725 kb)

Additional file 7: Figure S7. *Cx3cr1*Δ*Il1r1*-, *Slco1c1*Δ*Myd88*-, and *Cx3/Slc*Δ*Myd88*-mediated disruption of IL-1β signaling does not affect the sickness response. Twenty-four-hour profiles of telemetric and feeding data shows the stereotypical IL-1β-induced elevation in core body temperature (ΔCBT) and decrease in voluntary locomotor activity (VLA) and food intake (FI) in both control (fl/fl) and strain-specific knockout animals (KO). *Cx3cr1*Δ*Il1r1* (A-C), *Slco1c1*Δ*Myd88* (D-F), and *Cx3/Slc*Δ*Myd88* (G-I) mice all exhibit IL-1β-induced sickness responses. Regardless of genotype ΔCBT, VLA and FI were significantly different from their vehicle-treated counterparts for several hours following 10 ng IL-1β treatment (*p* < 0.05 for times below black (fl/fl) and red (KO) bars above traces in A-C). Gray boxes show dark phase, when mice are most active. All values shown are mean ± SEM for group sizes listed in the legend above A, D, and G. (TIFF 609 kb)

Additional file 8: Figure S8. *Cx3cr1*Δ*Myd88*-mediated disruption of IL-1β signaling exclusively in microglia does not affect sickness responses. Both *Cx3cr1*Δ*Myd88* (KO) and their Cre−, *Myd88* ^fl/fl^ littermates (fl/fl) exhibit IL-1β-induced sickness responses. Regardless of genotype IL-1β treatment caused a significant reduction in both overnight (6 pm–6 am) total food intake (FI; A) and change in body weight (ΔBW; B). All values shown are mean ± SEM for group sizes shown in legend. **p < 0.01. (TIFF 123 kb)

## Abbreviations

3V: Third ventricle
aCSF: Artificial cerebrospinal fluid
ANOVA: Analysis of variance
ARC/ME: Arcuate nucleus/median eminence
BBB: Blood-brain barrier
BW: Body weight
CBT: Core body temperature
Cd11b: Cluster of differentiation molecule 11b
Cd31: Cluster of differentiation 31
ChP: Choroid plexus
CNS: Central nervous system
COX-2: Cyclooxygenase-2
Cre: Cre recombinase
Cre-ERT2: Tamoxifen-inducible Cre recombinase
CSF: Cerebrospinal fluid
CVOs: Circumventricular organs
FI: Food intake
GFAP: Glial fibrillary acidic protein
HPA: Hypothalamic-pituitary-adrenal
ICV: Intracerebroventricular
IHC: Immunohistochemistry
*Il1r1*: Interleukin-1 receptor 1
Il1ra: Interleukin-1 receptor 1 antagonist
Il1rap: Interleukin-1 receptor accessory protein
IL-1β: Interleukin-1β
IR: Immunoreactivity
KO: Knockout
MnPOA: Median preoptic nucleus
Myd88: Myeloid differentiation primary response gene 88
NF-κB: Nuclear factor kappa-light-chain-enhancer of activated B cells
OVLT: Organum vasculosum lamina terminalis
PBS: Phosphate-buffered saline
PGE2: Prostaglandin E2
PGs: Prostaglandins
PVN: Paraventricular nucleus
SFO: Subfornical organ
*Slco1c1*: Solute carrier organic anion transporter family member 1C1
Stat3: Signal transducer and activator of transcription 3
TdT: Tandem-dimer tomato
TH: Thyroid hormone
Veh: Vehicle
VLA: Voluntary locomotor activity
YFP: Yellow fluorescent protein
