# Supplementary figures and images for: Interleukin-1β signaling in fenestrated capillaries is sufficient to trigger sickness responses in mice
Source: J Neuroinflammation. 2017 Nov 9;14:219. doi: 10.1186/s12974-017-0990-7 (PMC5680784; doi:10.1186/s12974-017-0990-7)

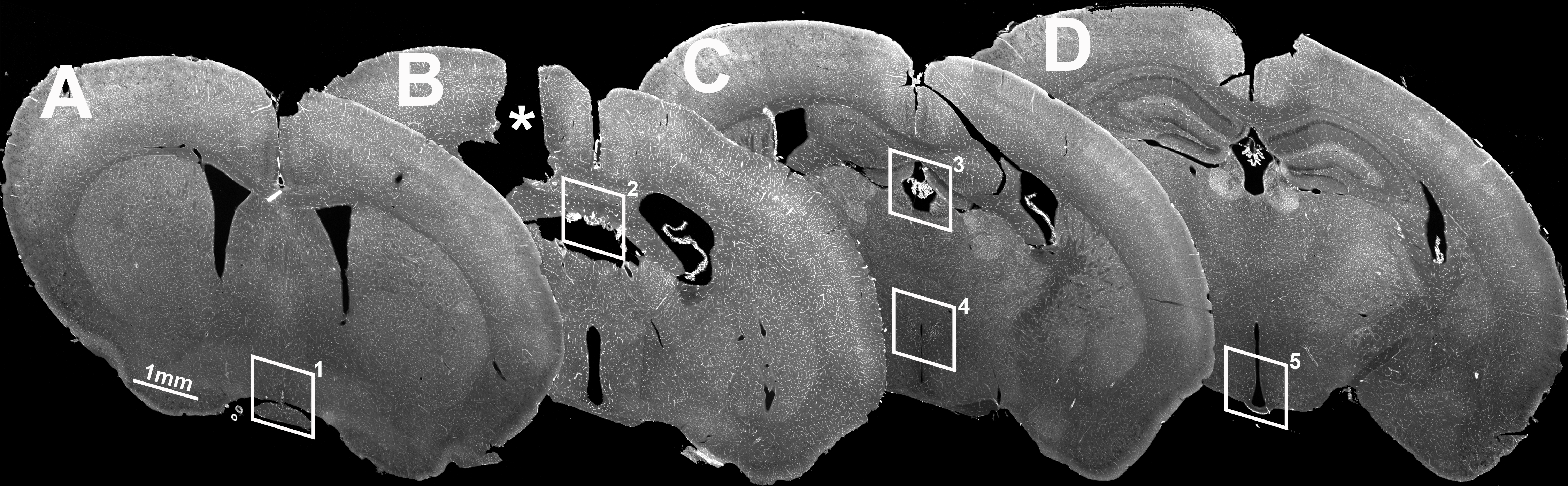

Supplement: Supplementary file 1 — Vascular heterogeneity in the mouse brain. (A-D) Cd31 IR in epifluorescent digital montages of representative brain sections from animals used for various experiments at bregma (A), bregma − 0.5 (B; asterisk shows the location where a cannula was placed to give access to the lateral ventricle), − 1.0 (C) and − 1.5 mm (D) according to the mouse brain atlas by Paxinos and Franklin [70]. Boxes indicate the five regions of interest shown in Fig. 1. Increased vascular density is evident in the organum vasculosum lamina terminalis (OVLT, 1), subfornical organ (SFO, 2), choroid plexus (ChP, 3), paraventricular nucleus (PVN, 4), and arcuate nucleus/median eminence (ARC/ME, 5). Scale bar = 1 mm. (TIFF 6352 kb) [file 12974_2017_990_MOESM1_ESM.tif]

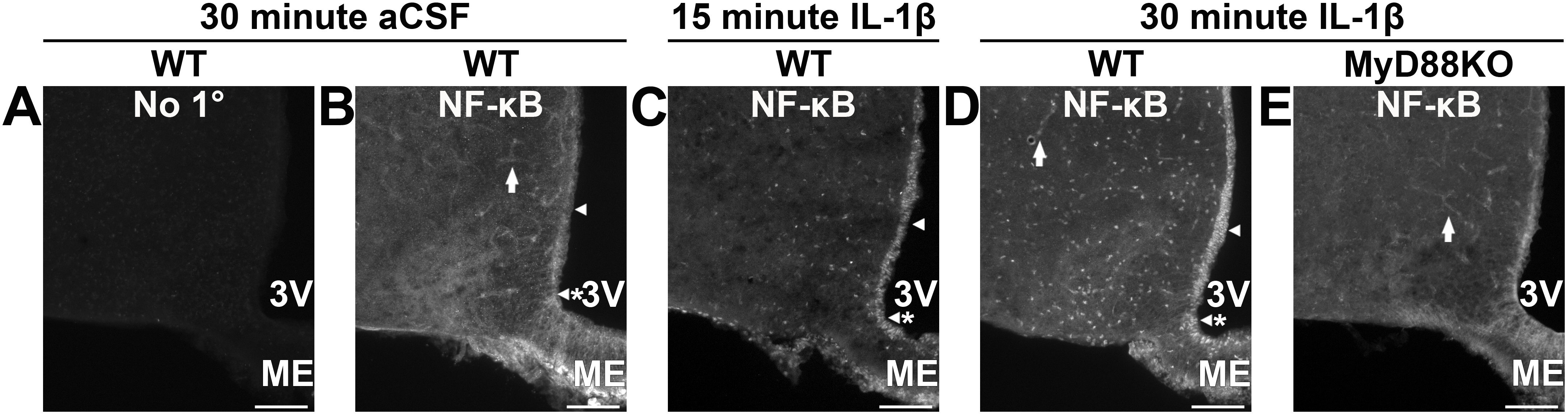

Supplement: Supplementary file 2 — IL-1β-induced nuclear localization of NF-κB requires Myd88. Representative epifluorescent images of the effects of central IL-1β. Omission of primary antibody (A) demonstrates that the vascular pattern of cytoplasmic immunoreactivity (IR, arrows) observed in vehicle (aCSF)-treated animals (B, n = 3) is specific to the NF-κB antibody. At the level of the arcuate nucleus/median eminence (ARC/ME), nuclear NF-κB IR is evident by 15 min (C, n = 4) and peaks around 30 min (D, n = 8) after ICV IL-1β treatment. Ependymal cells lining the third ventricle (cuboidal nuclei, arrowheads), tanycytes (columnar nuclei, asterisk), and endothelial cells (arrow in D) demonstrate nuclear NF-κB IR. As with vehicle treated animals, NF-κB IR remained cytoplasmic in IL-1β-treated Myd88KO (E, arrows, n = 3) animals at all times examined. 3V = third ventricle, ME = median eminence. Scale bars = 100 μm. (TIFF 3612 kb) [file 12974_2017_990_MOESM2_ESM.tif]

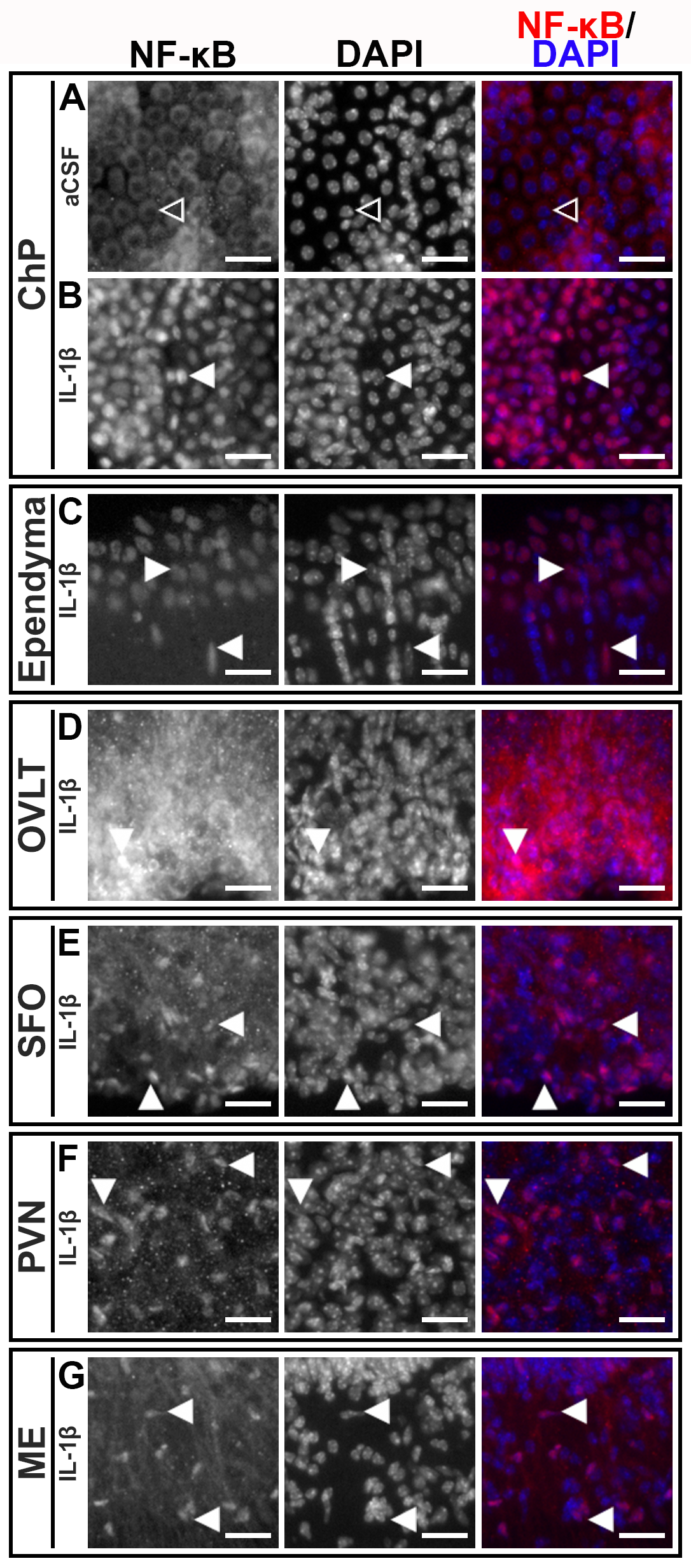

Supplement: Supplementary file 3 — DAPI labeling confirms IL-1β-induced NF-κB nuclear localization. Representative epifluorescent images of NF-κB immunoreactivity (IR; left column) and DAPI labeling of nuclear DNA (middle column) 30 min after treatment demonstrate the change in cellular localization caused by 10 ng intracerebroventricular (ICV) IL-1β. This effect is most obvious in the cuboidal cells of the choroid plexus (ChP), where NF-κB IR is found predominantly in the cytoplasm in sections from vehicle-treated animals (artificial cerebrospinal fluid, aCSF; open arrowhead in A) and concentrated in the nucleus of IL-1β-treated animals (arrowhead in B). Like the ChP, the ependymal cells (C) that form the barrier between the CSF and the brain consistently exhibit NF-κB nuclear localization, serving as an indicator that the animal has been exposed to IL-1β. While there are cells in all tissues, there that do not respond to IL-1β (DAPI-labeled blue nuclei that do not co-label with red NF-κB; right column), puncta of concentrated NF-κB IR overlap DAPI, indicating nuclear localization (filled arrowheads). This is true for all regions of the brain, including the organum vasculosum lamina terminalis (OVLT; D), the subfornical organ (SFO; E), the paraventricular nucleus (PVN; F), and median eminence (ME; G). The high cellular density of the brain, particularly within the OVLT, SFO, and PVN, makes it difficult to distinguish between different cells. Scale bars = 25 μm. (TIFF 2487 kb) [file 12974_2017_990_MOESM3_ESM.tif]

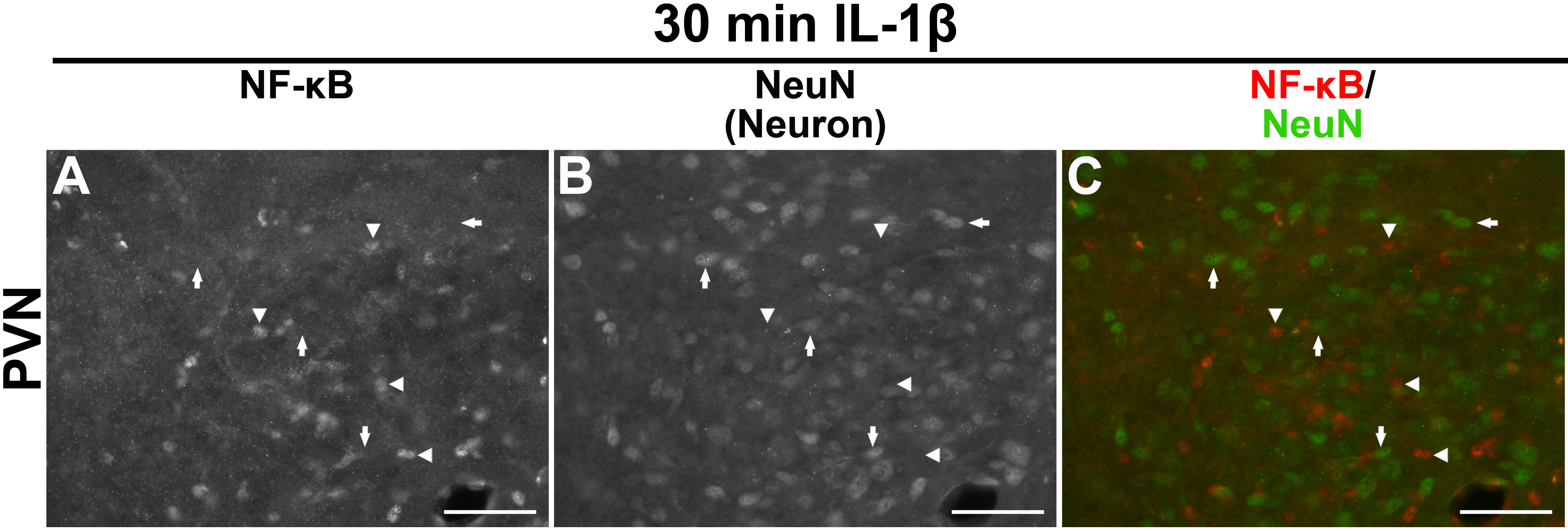

Supplement: Supplementary file 4 — Neurons in the PVN do not exhibit IL-1β-induced nuclear NF-κB. Representative epifluorescent images show that IL-1β causes nuclear localization of NF-κB (green) within the paraventricular nucleus (PVN; A, arrowheads). Despite a high density of neuronal nuclei (NeuN, red; B, arrows), there was no evidence of co-localization (C, n = 3). Scale bars = 50 μm. (TIFF 7122 kb) [file 12974_2017_990_MOESM4_ESM.tif]

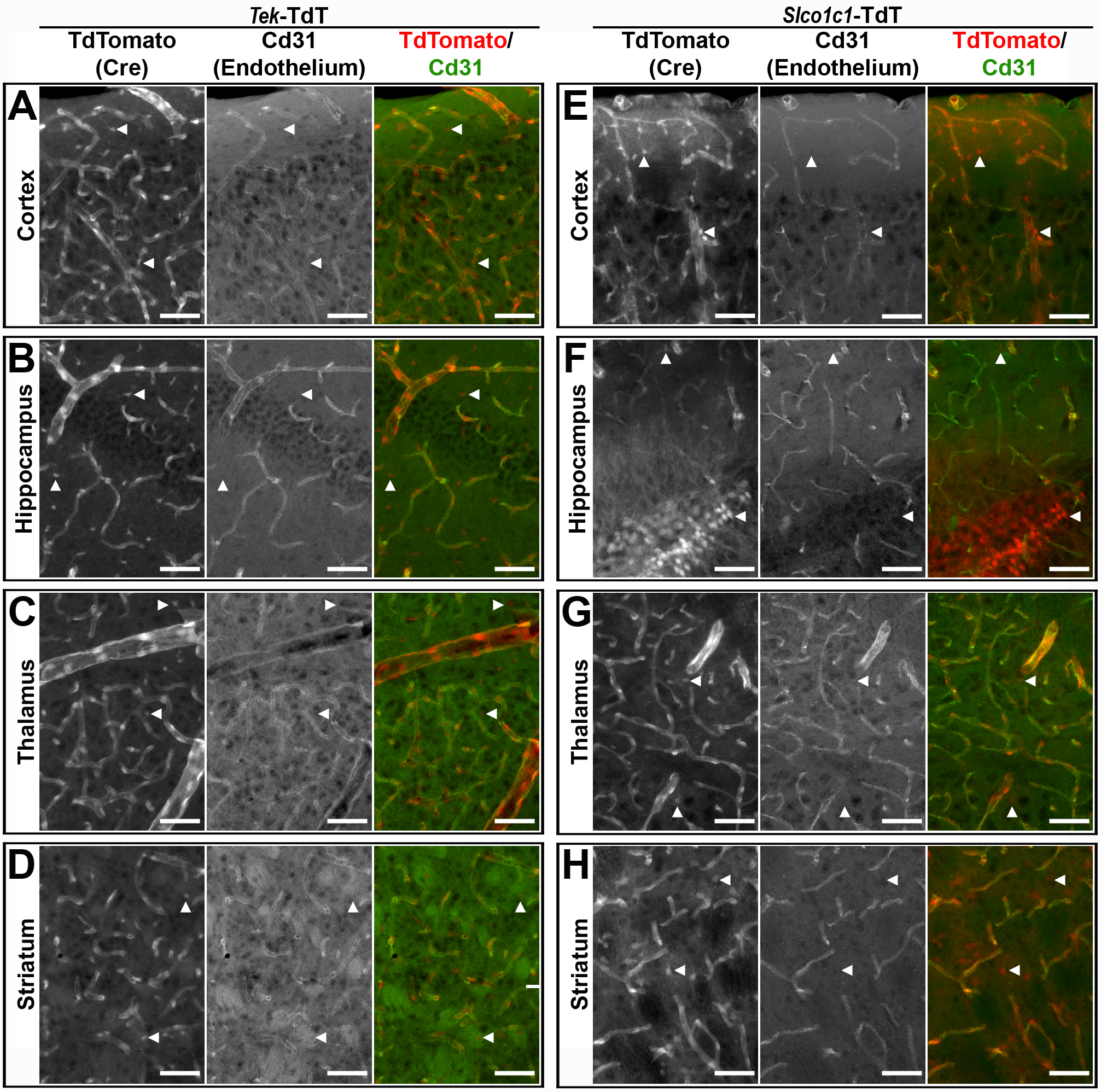

Supplement: Supplementary file 5 — Both Tek-Cre and Slco1c1-CreERT2 drive recombination in parenchymal endothelium. Representative epifluorescent images demonstrate that TdTomato (TdT, red) expression was present in all parenchymal endothelium (Cd31+, green) in both Tek-TdT (A-D, n = 4) and Slco1c1-TdT (E-H, n = 4) animals in all brain regions examined. Expression outside of blood vessels (arrowheads) was also present in both lines in all regions. Scale bars = 50 μm. (TIFF 4799 kb) [file 12974_2017_990_MOESM5_ESM.tif]

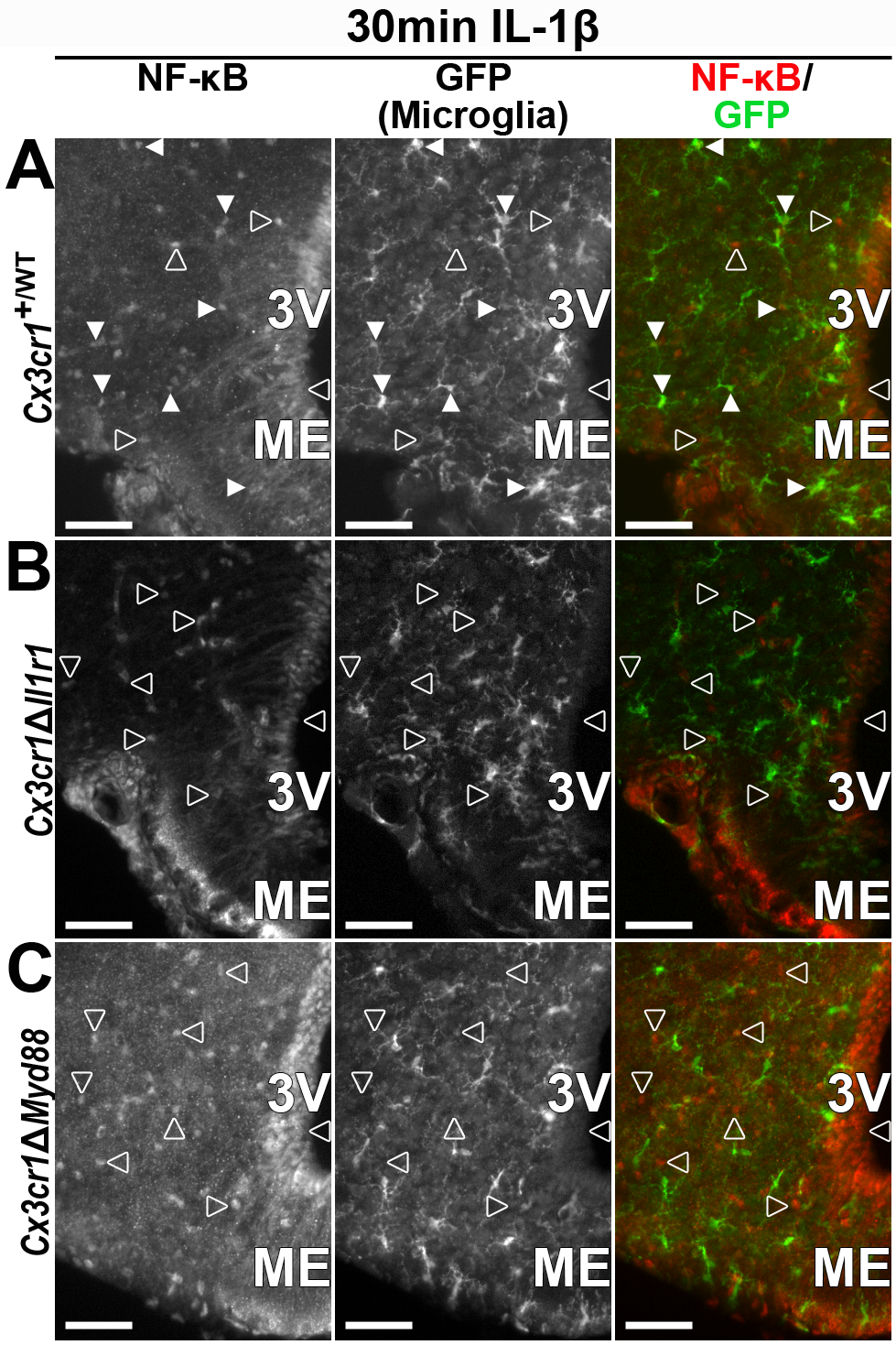

Supplement: Supplementary file 6 — Cx3cr1-CreERT2 causes genetic recombination exclusively in microglia. Representative epifluorescent images of NF-κB immunoreactivity (IR) 30 min after ICV IL-1β demonstrate Cx3cr1-CreERT2-mediated disruption of signaling in microglia when either the interleukin-1 receptor (Il1r1) or Myd88 is deleted. The Cx3cr1-CreERT2 transgene contains a sequence coding for YFP that allows for visualization of microglia using an anti-GFP antibody. In Cre + animals that do not have floxed Il1r1 or Myd88 (Cx3cr1 +/WT, n = 5), nuclear NF-κB is found in GFP+ microglia in the arcuate nucleus/median eminence (ARC/ME; co-expression denoted by filled arrowheads in A) and in cells that do not express GFP, including ependymal cells lining the third ventricle (3V; open arrowheads). In Cre + animals that are homozygous for floxed alleles of Il1r1 (Cx3cr1ΔIl1r1, B, n = 4) or Myd88 (Cx3cr1ΔMyd88, C, n = 3) nuclear NF-κB IR is mostly absent from GFP+ microglia. Nuclear NF-κB IR in cells that do not express GFP (open arrowheads in B and C) demonstrates that IL-1β signaling is not disrupted globally. Scale bars = 50 μm. (TIFF 1725 kb) [file 12974_2017_990_MOESM6_ESM.tif]

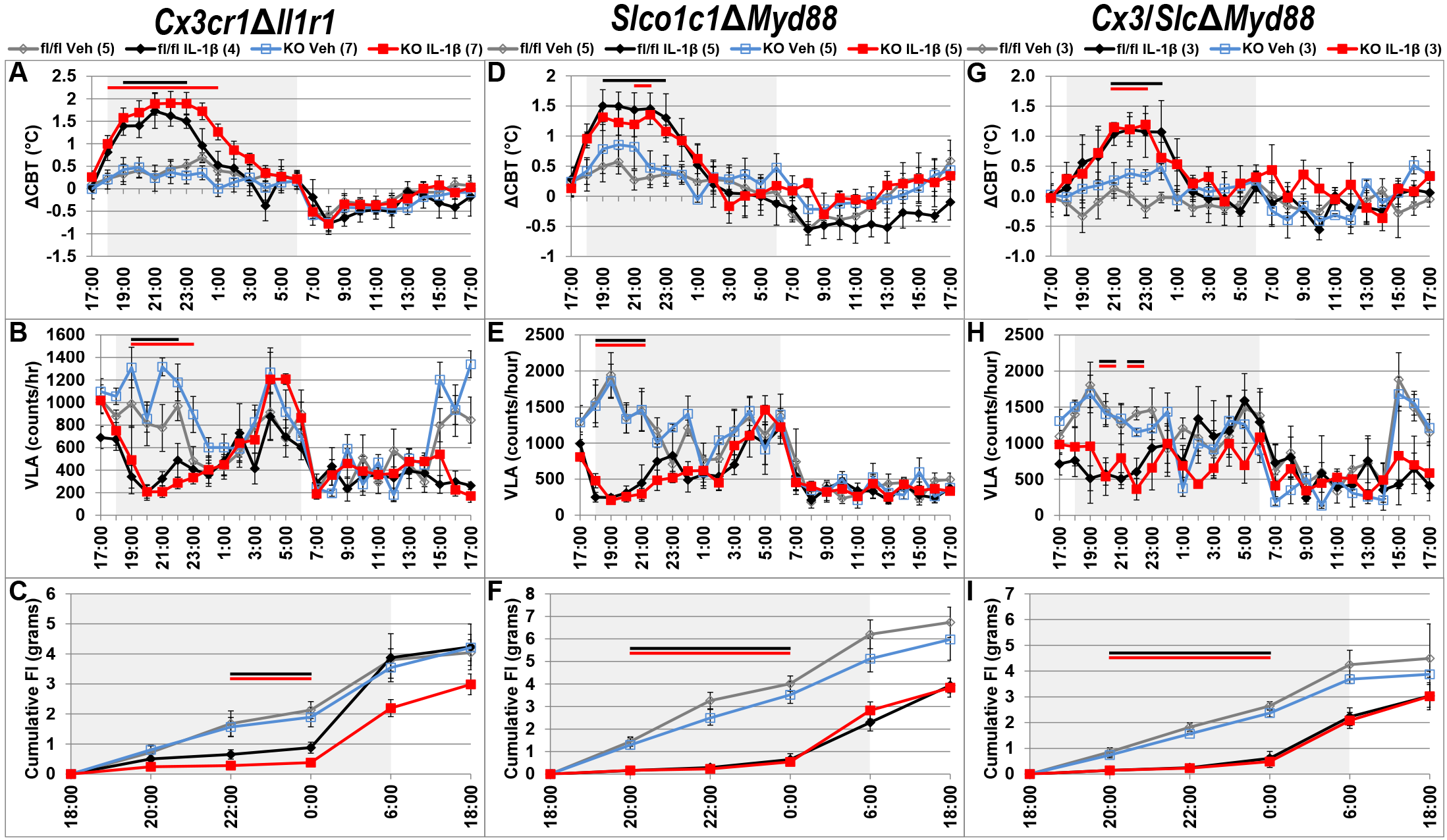

Supplement: Supplementary file 7 — Cx3cr1ΔIl1r1-, Slco1c1ΔMyd88-, and Cx3/SlcΔMyd88-mediated disruption of IL-1β signaling does not affect the sickness response. Twenty-four-hour profiles of telemetric and feeding data shows the stereotypical IL-1β-induced elevation in core body temperature (ΔCBT) and decrease in voluntary locomotor activity (VLA) and food intake (FI) in both control (fl/fl) and strain-specific knockout animals (KO). Cx3cr1ΔIl1r1 (A-C), Slco1c1ΔMyd88 (D-F), and Cx3/SlcΔMyd88 (G-I) mice all exhibit IL-1β-induced sickness responses. Regardless of genotype ΔCBT, VLA and FI were significantly different from their vehicle-treated counterparts for several hours following 10 ng IL-1β treatment (p < 0.05 for times below black (fl/fl) and red (KO) bars above traces in A-C). Gray boxes show dark phase, when mice are most active. All values shown are mean ± SEM for group sizes listed in the legend above A, D, and G. (TIFF 609 kb) [file 12974_2017_990_MOESM7_ESM.tif]

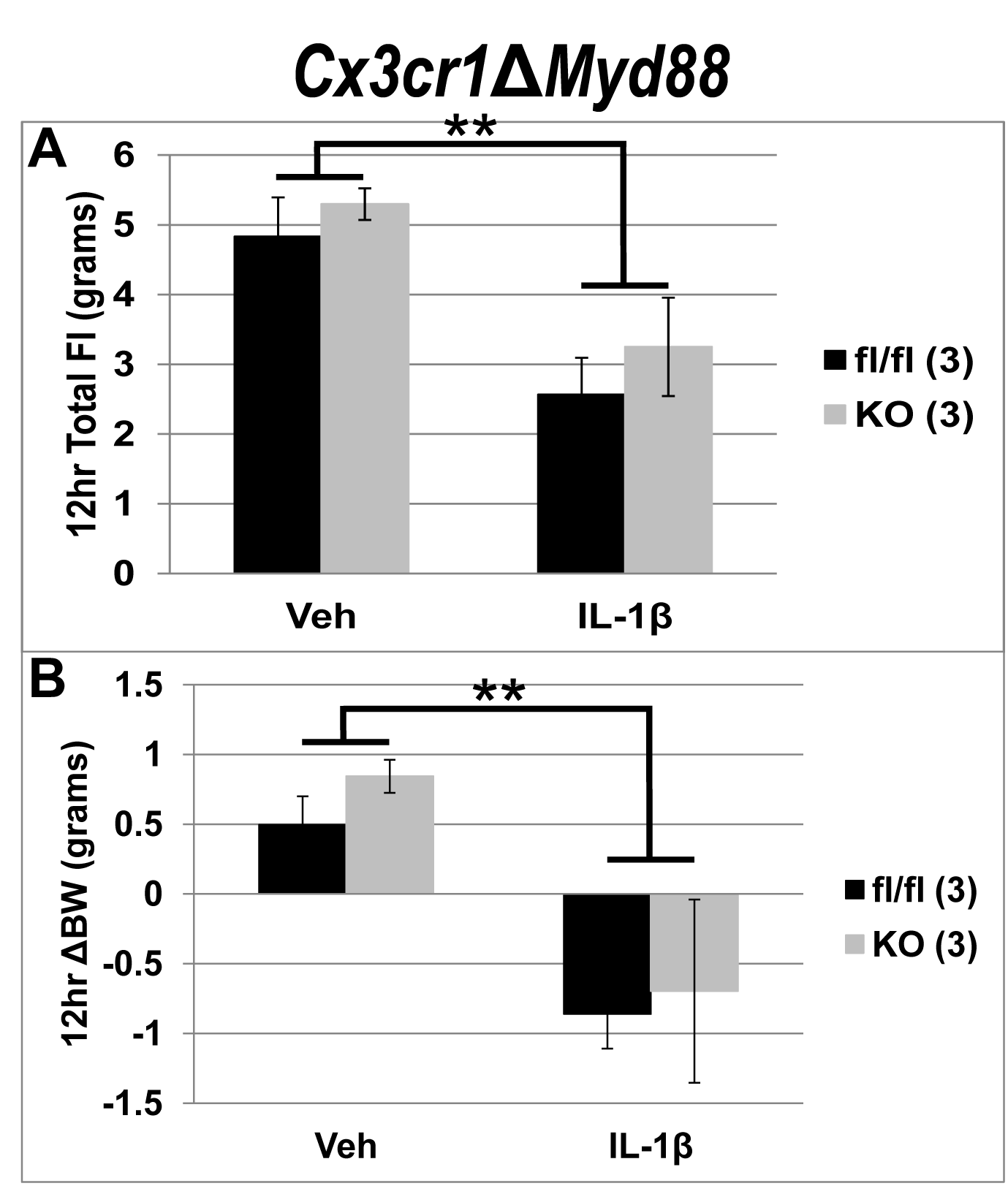

Supplement: Supplementary file 8 — Cx3cr1ΔMyd88-mediated disruption of IL-1β signaling exclusively in microglia does not affect sickness responses. Both Cx3cr1ΔMyd88 (KO) and their Cre−, Myd88 fl/fl littermates (fl/fl) exhibit IL-1β-induced sickness responses. Regardless of genotype IL-1β treatment caused a significant reduction in both overnight (6 pm–6 am) total food intake (FI; A) and change in body weight (ΔBW; B). All values shown are mean ± SEM for group sizes shown in legend. **p < 0.01. (TIFF 123 kb) [file 12974_2017_990_MOESM8_ESM.tif]
